# Supplementary material for: Centrifuge: rapid and sensitive classification of metagenomic sequences
Source: Genome Res. 2016 Dec;26(12):1721–9. doi: 10.1101/gr.210641.116 (PMC5131823; doi:10.1101/gr.210641.116)
Supplement: Supplemental Material [file supp_26_12_1721__index.html]

Centrifuge: rapid and sensitive classification of metagenomic sequences — Supplemental Material 

# Centrifuge: rapid and sensitive classification of metagenomic sequences

## Supplemental Material

- Supplemental\_Data\_S1.zip
- Supplemental\_Figure\_S1.docx
- Supplemental\_Figure\_S2.docx
- Supplemental\_Methods.docx
- Supplemental\_Table\_S1.docx
- Supplemental\_Table\_S2.xlsx
- Supplemental\_Table\_S3.xlsx
- Supplemental\_Table\_S4.xlsx
