# Supplementary material for: Centrifuge: rapid and sensitive classification of metagenomic sequences
Source: Genome Res. 2016 Dec;26(12):1721–9. doi: 10.1101/gr.210641.116 (PMC5131823; doi:10.1101/gr.210641.116)
Supplement: Supplemental Material [file supp_gr.210641.116_Supplemental_Data_S1.zip › centrifuge-centrifuge-genome-research/doc/footer.inc.html]

|  |  |
| --- | --- |
| This research was supported in part by NIH grants R01-LM06845 and R01-GM083873 and NSF grant CCF-0347992. | Administrator: Daehwan Kim. Design by David Herreman |
