# Supplementary material for: Centrifuge: rapid and sensitive classification of metagenomic sequences
Source: Genome Res. 2016 Dec;26(12):1721–9. doi: 10.1101/gr.210641.116 (PMC5131823; doi:10.1101/gr.210641.116)
Supplement: Supplemental Material [file supp_gr.210641.116_Supplemental_Data_S1.zip › centrifuge-centrifuge-genome-research/doc/manual.html]

HISAT Manual - 


# Table of Contents

- Introduction
  - What is HISAT?
- Obtaining Bowtie 2
  - Building from source
  - Adding to PATH
  - Reporting
    - Distinct alignments map a read to different places
    - Default mode: search for multiple alignments, report the best one
    - -k mode: search for one or more alignments, report each
  - Alignment summmary
  - Wrapper
  - Performance tuning
  - Command Line
    - Setting function options
    - Usage
    - Main arguments
    - Options
  - SAM output
- The `bowtie2-build` indexer
  - Command Line
    - Main arguments
    - Options
- The `bowtie2-inspect` index inspector
  - Command Line
    - Main arguments
    - Options
- Getting started with Bowtie 2: Lambda phage example
  - Indexing a reference genome
  - Aligning example reads
  - Paired-end example
  - Local alignment example
  - Using SAMtools/BCFtools downstream

# Introduction

## What is HISAT?

HISAT is an ultrafast and memory-efficient tool for aligning sequencing reads to long reference sequences. It is particularly good at aligning reads of about 50 up to 100s or 1,000s of characters to relatively long (e.g. mammalian) genomes. Bowtie 2 indexes the genome with an FM Index (based on the Burrows-Wheeler Transform or BWT) to keep its memory footprint small: for the human genome, its memory footprint is typically around 3.2 gigabytes of RAM. Bowtie 2 supports gapped, local, and paired-end alignment modes. Multiple processors can be used simultaneously to achieve greater alignment speed. Bowtie 2 outputs alignments in SAM format, enabling interoperation with a large number of other tools (e.g. SAMtools, GATK) that use SAM. Bowtie 2 is distributed under the GPLv3 license, and it runs on the command line under Windows, Mac OS X and Linux.

Bowtie 2 is often the first step in pipelines for comparative genomics, including for variation calling, ChIP-seq, RNA-seq, BS-seq. Bowtie 2 and Bowtie (also called "Bowtie 1" here) are also tightly integrated into some tools, including TopHat: a fast splice junction mapper for RNA-seq reads, Cufflinks: a tool for transcriptome assembly and isoform quantitiation from RNA-seq reads, Crossbow: a cloud-enabled software tool for analyzing reseuqncing data, and Myrna: a cloud-enabled software tool for aligning RNA-seq reads and measuring differential gene expression.

# Obtaining Bowtie 2

Download Bowtie 2 sources and binaries from the Download section of the Sourceforge site. Binaries are available for Intel architectures (`i386` and `x86_64`) running Linux, and Mac OS X. A 32-bit version is available for Windows. If you plan to compile Bowtie 2 yourself, make sure to get the source package, i.e., the filename that ends in "-source.zip".

## Building from source

Building Bowtie 2 from source requires a GNU-like environment with GCC, GNU Make and other basics. It should be possible to build Bowtie 2 on most vanilla Linux installations or on a Mac installation with Xcode installed. Bowtie 2 can also be built on Windows using Cygwin or MinGW (MinGW recommended). For a MinGW build the choice of what compiler is to be used is important since this will determine if a 32 or 64 bit code can be successfully compiled using it. If there is a need to generate both 32 and 64 bit on the same machine then a multilib MinGW has to be properly installed. MSYS, the zlib library, and depending on architecture pthreads library are also required. We are recommending a 64 bit build since it has some clear advantages in real life research problems. In order to simplify the MinGW setup it might be worth investigating popular MinGW personal builds since these are coming already prepared with most of the toolchains needed.

First, download the source package from the sourceforge site. Make sure you're getting the source package; the file downloaded should end in `-source.zip`. Unzip the file, change to the unzipped directory, and build the Bowtie 2 tools by running GNU `make` (usually with the command `make`, but sometimes with `gmake`) with no arguments. If building with MinGW, run `make` from the MSYS environment.

Bowtie 2 is using the multithreading software model in order to speed up execution times on SMP architectures where this is possible. On POSIX platforms (like linux, Mac OS, etc) it needs the pthread library. Although it is possible to use pthread library on non-POSIX platform like Windows, due to performance reasons bowtie 2 will try to use Windows native multithreading if possible.

## Adding to PATH

By adding your new Bowtie 2 directory to your PATH environment variable, you ensure that whenever you run `bowtie2`, `bowtie2-build` or `bowtie2-inspect` from the command line, you will get the version you just installed without having to specify the entire path. This is recommended for most users. To do this, follow your operating system's instructions for adding the directory to your PATH.

If you would like to install Bowtie 2 by copying the Bowtie 2 executable files to an existing directory in your PATH, make sure that you copy all the executables, including `bowtie2`, `bowtie2-align`, `bowtie2-build` and `bowtie2-inspect`.

## Reporting

The reporting mode governs how many alignments Bowtie 2 looks for, and how to report them. Bowtie 2 has three distinct reporting modes. The default reporting mode is similar to the default reporting mode of many other read alignment tools, including BWA. It is also similar to Bowtie 1's `-M` alignment mode.

In general, when we say that a read has an alignment, we mean that it has a valid alignment. When we say that a read has multiple alignments, we mean that it has multiple alignments that are valid and distinct from one another.

### Distinct alignments map a read to different places

Two alignments for the same individual read are "distinct" if they map the same read to different places. Specifically, we say that two alignments are distinct if there are no alignment positions where a particular read offset is aligned opposite a particular reference offset in both alignments with the same orientation. E.g. if the first alignment is in the forward orientation and aligns the read character at read offset 10 to the reference character at chromosome 3, offset 3,445,245, and the second alignment is also in the forward orientation and also aligns the read character at read offset 10 to the reference character at chromosome 3, offset 3,445,245, they are not distinct alignments.

Two alignments for the same pair are distinct if either the mate 1s in the two paired-end alignments are distinct or the mate 2s in the two alignments are distinct or both.

### Default mode: search for multiple alignments, report the best one

By default, Bowtie 2 searches for distinct, valid alignments for each read. When it finds a valid alignment, it generally will continue to look for alignments that are nearly as good or better. It will eventually stop looking, either because it exceeded a limit placed on search effort (see [`-D`] and `-R`) or because it already knows all it needs to know to report an alignment. Information from the best alignments are used to estimate mapping quality (the `MAPQ` SAM field) and to set SAM optional fields, such as `AS:i` and `XS:i`. Bowtie 2 does not gaurantee that the alignment reported is the best possible in terms of alignment score.

See also: [`-D`], which puts an upper limit on the number of dynamic programming problems (i.e. seed extensions) that can "fail" in a row before Bowtie 2 stops searching. Increasing [`-D`] makes Bowtie 2 slower, but increases the likelihood that it will report the correct alignment for a read that aligns many places.

See also: `-R`, which sets the maximum number of times Bowtie 2 will "re-seed" when attempting to align a read with repetitive seeds. Increasing `-R` makes Bowtie 2 slower, but increases the likelihood that it will report the correct alignment for a read that aligns many places.

### -k mode: search for one or more alignments, report each

In `-k` mode, Bowtie 2 searches for up to N distinct, valid alignments for each read, where N equals the integer specified with the `-k` parameter. That is, if `-k 2` is specified, Bowtie 2 will search for at most 2 distinct alignments. It reports all alignments found, in descending order by alignment score. The alignment score for a paired-end alignment equals the sum of the alignment scores of the individual mates. Each reported read or pair alignment beyond the first has the SAM 'secondary' bit (which equals 256) set in its FLAGS field. See the SAM specification for details.

Bowtie 2 does not "find" alignments in any specific order, so for reads that have more than N distinct, valid alignments, Bowtie 2 does not gaurantee that the N alignments reported are the best possible in terms of alignment score. Still, this mode can be effective and fast in situations where the user cares more about whether a read aligns (or aligns a certain number of times) than where exactly it originated.

## Alignment summmary

When Bowtie 2 finishes running, it prints messages summarizing what happened. These messages are printed to the "standard error" ("stderr") filehandle. For datasets consisting of unpaired reads, the summary might look like this:

```
20000 reads; of these:
  20000 (100.00%) were unpaired; of these:
    1247 (6.24%) aligned 0 times
    18739 (93.69%) aligned exactly 1 time
    14 (0.07%) aligned >1 times
93.77% overall alignment rate
```

For datasets consisting of pairs, the summary might look like this:

```
10000 reads; of these:
  10000 (100.00%) were paired; of these:
    650 (6.50%) aligned concordantly 0 times
    8823 (88.23%) aligned concordantly exactly 1 time
    527 (5.27%) aligned concordantly >1 times
    ----
    650 pairs aligned concordantly 0 times; of these:
      34 (5.23%) aligned discordantly 1 time
    ----
    616 pairs aligned 0 times concordantly or discordantly; of these:
      1232 mates make up the pairs; of these:
        660 (53.57%) aligned 0 times
        571 (46.35%) aligned exactly 1 time
        1 (0.08%) aligned >1 times
96.70% overall alignment rate
```

The indentation indicates how subtotals relate to totals.

## Wrapper

The `bowtie2` executable is actually a Perl wrapper script that calls the compiled `bowtie2-align` binary. It is recommended that you always run the `bowtie2` wrapper and not run `bowtie2-align` directly.

## Performance tuning

1. Use 64-bit version if possible

   The 64-bit version of Bowtie 2 is faster than the 32-bit version, owing to its use of 64-bit arithmetic. If possible, download the 64-bit binaries for Bowtie 2 and run on a 64-bit computer. If you are building Bowtie 2 from sources, you may need to pass the `-m64` option to `g++` to compile the 64-bit version; you can do this by including `BITS=64` in the arguments to the `make` command; e.g.: `make BITS=64 bowtie2`. To determine whether your version of bowtie is 64-bit or 32-bit, run `bowtie2 --version`.
2. If your computer has multiple processors/cores, use `-p`

   The `-p` option causes Bowtie 2 to launch a specified number of parallel search threads. Each thread runs on a different processor/core and all threads find alignments in parallel, increasing alignment throughput by approximately a multiple of the number of threads (though in practice, speedup is somewhat worse than linear).

## Command Line

### Setting function options

Some Bowtie 2 options specify a function rather than an individual number or setting. In these cases the user specifies three parameters: (a) a function type `F`, (b) a constant term `B`, and (c) a coefficient `A`. The available function types are constant (`C`), linear (`L`), square-root (`S`), and natural log (`G`). The parameters are specified as `F,B,A` - that is, the function type, the constant term, and the coefficient are separated by commas with no whitespace. The constant term and coefficient may be negative and/or floating-point numbers.

For example, if the function specification is `L,-0.4,-0.6`, then the function defined is:

```
f(x) = -0.4 + -0.6 * x
```

If the function specification is `G,1,5.4`, then the function defined is:

```
f(x) = 1.0 + 5.4 * ln(x)
```

See the documentation for the option in question to learn what the parameter `x` is for. For example, in the case if the `--score-min` option, the function `f(x)` sets the minimum alignment score necessary for an alignment to be considered valid, and `x` is the read length.

### Usage

```
bowtie2 [options]* -x <bt2-idx> {-1 <m1> -2 <m2> | -U <r>} -S [<hit>]
```

### Main arguments

|  |  |
| --- | --- |
| ``` -x <bt2-idx> ``` | The basename of the index for the reference genome. The basename is the name of any of the index files up to but not including the final `.1.bt2` / `.rev.1.bt2` / etc. `bowtie2` looks for the specified index first in the current directory, then in the directory specified in the `BOWTIE2_INDEXES` environment variable. |
| ``` -1 <m1> ``` | Comma-separated list of files containing mate 1s (filename usually includes `_1`), e.g. `-1 flyA_1.fq,flyB_1.fq`. Sequences specified with this option must correspond file-for-file and read-for-read with those specified in `<m2>`. Reads may be a mix of different lengths. If `-` is specified, `bowtie2` will read the mate 1s from the "standard in" or "stdin" filehandle. |
| ``` -2 <m2> ``` | Comma-separated list of files containing mate 2s (filename usually includes `_2`), e.g. `-2 flyA_2.fq,flyB_2.fq`. Sequences specified with this option must correspond file-for-file and read-for-read with those specified in `<m1>`. Reads may be a mix of different lengths. If `-` is specified, `bowtie2` will read the mate 2s from the "standard in" or "stdin" filehandle. |
| ``` -U <r> ``` | Comma-separated list of files containing unpaired reads to be aligned, e.g. `lane1.fq,lane2.fq,lane3.fq,lane4.fq`. Reads may be a mix of different lengths. If `-` is specified, `bowtie2` gets the reads from the "standard in" or "stdin" filehandle. |
| ``` -S <hit> ``` | File to write SAM alignments to. By default, alignments are written to the "standard out" or "stdout" filehandle (i.e. the console). |

### Options

#### Input options

|  |  |
| --- | --- |
| ``` -q ``` | Reads (specified with `<m1>`, `<m2>`, `<s>`) are FASTQ files. FASTQ files usually have extension `.fq` or `.fastq`. FASTQ is the default format. See also: `--solexa-quals` and `--int-quals`. |
| ``` --qseq ``` | Reads (specified with `<m1>`, `<m2>`, `<s>`) are QSEQ files. QSEQ files usually end in `_qseq.txt`. See also: `--solexa-quals` and `--int-quals`. |
| ``` -f ``` | Reads (specified with `<m1>`, `<m2>`, `<s>`) are FASTA files. FASTA files usually have extension `.fa`, `.fasta`, `.mfa`, `.fna` or similar. FASTA files do not have a way of specifying quality values, so when `-f` is set, the result is as if `--ignore-quals` is also set. |
| ``` -r ``` | Reads (specified with `<m1>`, `<m2>`, `<s>`) are files with one input sequence per line, without any other information (no read names, no qualities). When `-r` is set, the result is as if `--ignore-quals` is also set. |
| ``` -c ``` | The read sequences are given on command line. I.e. `<m1>`, `<m2>` and `<singles>` are comma-separated lists of reads rather than lists of read files. There is no way to specify read names or qualities, so `-c` also implies `--ignore-quals`. |
| ``` -s/--skip <int> ``` | Skip (i.e. do not align) the first `<int>` reads or pairs in the input. |
| ``` -u/--qupto <int> ``` | Align the first `<int>` reads or read pairs from the input (after the `-s`/`--skip` reads or pairs have been skipped), then stop. Default: no limit. |
| ``` -5/--trim5 <int> ``` | Trim `<int>` bases from 5' (left) end of each read before alignment (default: 0). |
| ``` -3/--trim3 <int> ``` | Trim `<int>` bases from 3' (right) end of each read before alignment (default: 0). |
| ``` --phred33 ``` | Input qualities are ASCII chars equal to the Phred quality plus 33. This is also called the "Phred+33" encoding, which is used by the very latest Illumina pipelines. |
| ``` --phred64 ``` | Input qualities are ASCII chars equal to the Phred quality plus 64. This is also called the "Phred+64" encoding. |
| ``` --solexa-quals ``` | Convert input qualities from Solexa (which can be negative) to Phred (which can't). This scheme was used in older Illumina GA Pipeline versions (prior to 1.3). Default: off. |
| ``` --int-quals ``` | Quality values are represented in the read input file as space-separated ASCII integers, e.g., `40 40 30 40`..., rather than ASCII characters, e.g., `II?I`.... Integers are treated as being on the Phred quality scale unless `--solexa-quals` is also specified. Default: off. |

#### Alignment options

|  |  |
| --- | --- |
| ``` --n-ceil <func> ``` | Sets a function governing the maximum number of ambiguous characters (usually `N`s and/or `.`s) allowed in a read as a function of read length. For instance, specifying `-L,0,0.15` sets the N-ceiling function `f` to `f(x) = 0 + 0.15 * x`, where x is the read length. See also: [setting function options]. Reads exceeding this ceiling are filtered out. Default: `L,0,0.15`. |
| ``` --ignore-quals ``` | When calculating a mismatch penalty, always consider the quality value at the mismatched position to be the highest possible, regardless of the actual value. I.e. input is treated as though all quality values are high. This is also the default behavior when the input doesn't specify quality values (e.g. in `-f`, `-r`, or `-c` modes). |
| ``` --nofw/--norc ``` | If `--nofw` is specified, `bowtie2` will not attempt to align unpaired reads to the forward (Watson) reference strand. If `--norc` is specified, `bowtie2` will not attempt to align unpaired reads against the reverse-complement (Crick) reference strand. In paired-end mode, `--nofw` and `--norc` pertain to the fragments; i.e. specifying `--nofw` causes `bowtie2` to explore only those paired-end configurations corresponding to fragments from the reverse-complement (Crick) strand. Default: both strands enabled. |
| ``` --end-to-end ``` | In this mode, Bowtie 2 requires that the entire read align from one end to the other, without any trimming (or "soft clipping") of characters from either end. The match bonus `--ma` always equals 0 in this mode, so all alignment scores are less than or equal to 0, and the greatest possible alignment score is 0. This is mutually exclusive with `--local`. `--end-to-end` is the default mode. |
| ``` --local ``` | In this mode, Bowtie 2 does not require that the entire read align from one end to the other. Rather, some characters may be omitted ("soft clipped") from the ends in order to achieve the greatest possible alignment score. The match bonus `--ma` is used in this mode, and the best possible alignment score is equal to the match bonus (`--ma`) times the length of the read. Specifying `--local` and one of the presets (e.g. `--local --very-fast`) is equivalent to specifying the local version of the preset (`--very-fast-local`). This is mutually exclusive with `--end-to-end`. `--end-to-end` is the default mode. |

#### Scoring options

|  |  |
| --- | --- |
| ``` --ma <int> ``` | Sets the match bonus. In `--local` mode `<int>` is added to the alignment score for each position where a read character aligns to a reference character and the characters match. Not used in `--end-to-end` mode. Default: 2. |
| ``` --mp MX,MN ``` | Sets the maximum (`MX`) and minimum (`MN`) mismatch penalties, both integers. A number less than or equal to `MX` and greater than or equal to `MN` is subtracted from the alignment score for each position where a read character aligns to a reference character, the characters do not match, and neither is an `N`. If `--ignore-quals` is specified, the number subtracted quals `MX`. Otherwise, the number subtracted is `MN + floor( (MX-MN)(MIN(Q, 40.0)/40.0) )` where Q is the Phred quality value. Default: `MX` = 6, `MN` = 2. |
| ``` --np <int> ``` | Sets penalty for positions where the read, reference, or both, contain an ambiguous character such as `N`. Default: 1. |
| ``` --rdg <int1>,<int2> ``` | Sets the read gap open (`<int1>`) and extend (`<int2>`) penalties. A read gap of length N gets a penalty of `<int1>` + N \* `<int2>`. Default: 5, 3. |
| ``` --rfg <int1>,<int2> ``` | Sets the reference gap open (`<int1>`) and extend (`<int2>`) penalties. A reference gap of length N gets a penalty of `<int1>` + N \* `<int2>`. Default: 5, 3. |
| ``` --score-min <func> ``` | Sets a function governing the minimum alignment score needed for an alignment to be considered "valid" (i.e. good enough to report). This is a function of read length. For instance, specifying `L,0,-0.6` sets the minimum-score function `f` to `f(x) = 0 + -0.6 * x`, where `x` is the read length. See also: [setting function options]. The default in `--end-to-end` mode is `L,-0.6,-0.6` and the default in `--local` mode is `G,20,8`. |

#### Reporting options

|  |  |
| --- | --- |
| ``` -k <int> ``` | By default, `bowtie2` searches for distinct, valid alignments for each read. When it finds a valid alignment, it continues looking for alignments that are nearly as good or better. The best alignment found is reported (randomly selected from among best if tied). Information about the best alignments is used to estimate mapping quality and to set SAM optional fields, such as `AS:i` and `XS:i`.  When `-k` is specified, however, `bowtie2` behaves differently. Instead, it searches for at most `<int>` distinct, valid alignments for each read. The search terminates when it can't find more distinct valid alignments, or when it finds `<int>`, whichever happens first. All alignments found are reported in descending order by alignment score. The alignment score for a paired-end alignment equals the sum of the alignment scores of the individual mates. Each reported read or pair alignment beyond the first has the SAM 'secondary' bit (which equals 256) set in its FLAGS field. For reads that have more than `<int>` distinct, valid alignments, `bowtie2` does not gaurantee that the `<int>` alignments reported are the best possible in terms of alignment score. `-k` is mutually exclusive with `-a`.  Note: Bowtie 2 is not designed with large values for `-k` in mind, and when aligning reads to long, repetitive genomes large `-k` can be very, very slow. |
| ``` -a ``` | Like `-k` but with no upper limit on number of alignments to search for. `-a` is mutually exclusive with `-k`.  Note: Bowtie 2 is not designed with `-a` mode in mind, and when aligning reads to long, repetitive genomes this mode can be very, very slow. |

#### Paired-end options

|  |  |
| --- | --- |
| ``` -I/--minins <int> ``` | The minimum fragment length for valid paired-end alignments. E.g. if `-I 60` is specified and a paired-end alignment consists of two 20-bp alignments in the appropriate orientation with a 20-bp gap between them, that alignment is considered valid (as long as `-X` is also satisfied). A 19-bp gap would not be valid in that case. If trimming options `-3` or `-5` are also used, the `-I` constraint is applied with respect to the untrimmed mates.  The larger the difference between `-I` and `-X`, the slower Bowtie 2 will run. This is because larger differences bewteen `-I` and `-X` require that Bowtie 2 scan a larger window to determine if a concordant alignment exists. For typical fragment length ranges (200 to 400 nucleotides), Bowtie 2 is very efficient.  Default: 0 (essentially imposing no minimum) |
| ``` -X/--maxins <int> ``` | The maximum fragment length for valid paired-end alignments. E.g. if `-X 100` is specified and a paired-end alignment consists of two 20-bp alignments in the proper orientation with a 60-bp gap between them, that alignment is considered valid (as long as `-I` is also satisfied). A 61-bp gap would not be valid in that case. If trimming options `-3` or `-5` are also used, the `-X` constraint is applied with respect to the untrimmed mates, not the trimmed mates.  The larger the difference between `-I` and `-X`, the slower Bowtie 2 will run. This is because larger differences bewteen `-I` and `-X` require that Bowtie 2 scan a larger window to determine if a concordant alignment exists. For typical fragment length ranges (200 to 400 nucleotides), Bowtie 2 is very efficient.  Default: 500. |
| ``` --fr/--rf/--ff ``` | The upstream/downstream mate orientations for a valid paired-end alignment against the forward reference strand. E.g., if `--fr` is specified and there is a candidate paired-end alignment where mate 1 appears upstream of the reverse complement of mate 2 and the fragment length constraints (`-I` and `-X`) are met, that alignment is valid. Also, if mate 2 appears upstream of the reverse complement of mate 1 and all other constraints are met, that too is valid. `--rf` likewise requires that an upstream mate1 be reverse-complemented and a downstream mate2 be forward-oriented. `--ff` requires both an upstream mate 1 and a downstream mate 2 to be forward-oriented. Default: `--fr` (appropriate for Illumina's Paired-end Sequencing Assay). |
| ``` --no-mixed ``` | By default, when `bowtie2` cannot find a concordant or discordant alignment for a pair, it then tries to find alignments for the individual mates. This option disables that behavior. |
| ``` --no-discordant ``` | By default, `bowtie2` looks for discordant alignments if it cannot find any concordant alignments. A discordant alignment is an alignment where both mates align uniquely, but that does not satisfy the paired-end constraints (`--fr`/`--rf`/`--ff`, `-I`, `-X`). This option disables that behavior. |
| ``` --dovetail ``` | If the mates "dovetail", that is if one mate alignment extends past the beginning of the other such that the wrong mate begins upstream, consider that to be concordant. See also: Mates can overlap, contain or dovetail each other. Default: mates cannot dovetail in a concordant alignment. |
| ``` --no-contain ``` | If one mate alignment contains the other, consider that to be non-concordant. See also: Mates can overlap, contain or dovetail each other. Default: a mate can contain the other in a concordant alignment. |
| ``` --no-overlap ``` | If one mate alignment overlaps the other at all, consider that to be non-concordant. See also: Mates can overlap, contain or dovetail each other. Default: mates can overlap in a concordant alignment. |

#### Output options

|  |  |
| --- | --- |
| ``` -t/--time ``` | Print the wall-clock time required to load the index files and align the reads. This is printed to the "standard error" ("stderr") filehandle. Default: off. |
| ``` --un <path> --un-gz <path> --un-bz2 <path> ``` | Write unpaired reads that fail to align to file at `<path>`. These reads correspond to the SAM records with the FLAGS `0x4` bit set and neither the `0x40` nor `0x80` bits set. If `--un-gz` is specified, output will be gzip compressed. If `--un-bz2` is specified, output will be bzip2 compressed. Reads written in this way will appear exactly as they did in the input file, without any modification (same sequence, same name, same quality string, same quality encoding). Reads will not necessarily appear in the same order as they did in the input. |
| ``` --al <path> --al-gz <path> --al-bz2 <path> ``` | Write unpaired reads that align at least once to file at `<path>`. These reads correspond to the SAM records with the FLAGS `0x4`, `0x40`, and `0x80` bits unset. If `--al-gz` is specified, output will be gzip compressed. If `--al-bz2` is specified, output will be bzip2 compressed. Reads written in this way will appear exactly as they did in the input file, without any modification (same sequence, same name, same quality string, same quality encoding). Reads will not necessarily appear in the same order as they did in the input. |
| ``` --un-conc <path> --un-conc-gz <path> --un-conc-bz2 <path> ``` | Write paired-end reads that fail to align concordantly to file(s) at `<path>`. These reads correspond to the SAM records with the FLAGS `0x4` bit set and either the `0x40` or `0x80` bit set (depending on whether it's mate #1 or #2). `.1` and `.2` strings are added to the filename to distinguish which file contains mate #1 and mate #2. If a percent symbol, `%`, is used in `<path>`, the percent symbol is replaced with `1` or `2` to make the per-mate filenames. Otherwise, `.1` or `.2` are added before the final dot in `<path>` to make the per-mate filenames. Reads written in this way will appear exactly as they did in the input files, without any modification (same sequence, same name, same quality string, same quality encoding). Reads will not necessarily appear in the same order as they did in the inputs. |
| ``` --al-conc <path> --al-conc-gz <path> --al-conc-bz2 <path> ``` | Write paired-end reads that align concordantly at least once to file(s) at `<path>`. These reads correspond to the SAM records with the FLAGS `0x4` bit unset and either the `0x40` or `0x80` bit set (depending on whether it's mate #1 or #2). `.1` and `.2` strings are added to the filename to distinguish which file contains mate #1 and mate #2. If a percent symbol, `%`, is used in `<path>`, the percent symbol is replaced with `1` or `2` to make the per-mate filenames. Otherwise, `.1` or `.2` are added before the final dot in `<path>` to make the per-mate filenames. Reads written in this way will appear exactly as they did in the input files, without any modification (same sequence, same name, same quality string, same quality encoding). Reads will not necessarily appear in the same order as they did in the inputs. |
| ``` --quiet ``` | Print nothing besides alignments and serious errors. |
| ``` --met-file <path> ``` | Write `bowtie2` metrics to file `<path>`. Having alignment metric can be useful for debugging certain problems, especially performance issues. See also: `--met`. Default: metrics disabled. |
| ``` --met-stderr <path> ``` | Write `bowtie2` metrics to the "standard error" ("stderr") filehandle. This is not mutually exclusive with `--met-file`. Having alignment metric can be useful for debugging certain problems, especially performance issues. See also: `--met`. Default: metrics disabled. |
| ``` --met <int> ``` | Write a new `bowtie2` metrics record every `<int>` seconds. Only matters if either `--met-stderr` or `--met-file` are specified. Default: 1. |

#### SAM options

|  |  |
| --- | --- |
| ``` --no-unal ``` | Suppress SAM records for reads that failed to align. |
| ``` --no-hd ``` | Suppress SAM header lines (starting with `@`). |
| ``` --no-sq ``` | Suppress `@SQ` SAM header lines. |
| ``` --rg-id <text> ``` | Set the read group ID to `<text>`. This causes the SAM `@RG` header line to be printed, with `<text>` as the value associated with the `ID:` tag. It also causes the `RG:Z:` extra field to be attached to each SAM output record, with value set to `<text>`. |
| ``` --rg <text> ``` | Add `<text>` (usually of the form `TAG:VAL`, e.g. `SM:Pool1`) as a field on the `@RG` header line. Note: in order for the `@RG` line to appear, `--rg-id` must also be specified. This is because the `ID` tag is required by the SAM Spec. Specify `--rg` multiple times to set multiple fields. See the SAM Spec for details about what fields are legal. |
| ``` --omit-sec-seq ``` | When printing secondary alignments, Bowtie 2 by default will write out the `SEQ` and `QUAL` strings. Specifying this option causes Bowtie 2 to print an asterix in those fields instead. |

#### Performance options

|  |  |
| --- | --- |
| ``` -o/--offrate <int> ``` | Override the offrate of the index with `<int>`. If `<int>` is greater than the offrate used to build the index, then some row markings are discarded when the index is read into memory. This reduces the memory footprint of the aligner but requires more time to calculate text offsets. `<int>` must be greater than the value used to build the index. |
| ``` -p/--threads NTHREADS ``` | Launch `NTHREADS` parallel search threads (default: 1). Threads will run on separate processors/cores and synchronize when parsing reads and outputting alignments. Searching for alignments is highly parallel, and speedup is close to linear. Increasing `-p` increases Bowtie 2's memory footprint. E.g. when aligning to a human genome index, increasing `-p` from 1 to 8 increases the memory footprint by a few hundred megabytes. This option is only available if `bowtie` is linked with the `pthreads` library (i.e. if `BOWTIE_PTHREADS=0` is not specified at build time). |
| ``` --reorder ``` | Guarantees that output SAM records are printed in an order corresponding to the order of the reads in the original input file, even when `-p` is set greater than 1. Specifying `--reorder` and setting `-p` greater than 1 causes Bowtie 2 to run somewhat slower and use somewhat more memory then if `--reorder` were not specified. Has no effect if `-p` is set to 1, since output order will naturally correspond to input order in that case. |
| ``` --mm ``` | Use memory-mapped I/O to load the index, rather than typical file I/O. Memory-mapping allows many concurrent `bowtie` processes on the same computer to share the same memory image of the index (i.e. you pay the memory overhead just once). This facilitates memory-efficient parallelization of `bowtie` in situations where using `-p` is not possible or not preferable. |

#### Other options

|  |  |
| --- | --- |
| ``` --qc-filter ``` | Filter out reads for which the QSEQ filter field is non-zero. Only has an effect when read format is `--qseq`. Default: off. |
| ``` --seed <int> ``` | Use `<int>` as the seed for pseudo-random number generator. Default: 0. |
| ``` --non-deterministic ``` | Normally, Bowtie 2 re-initializes its pseudo-random generator for each read. It seeds the generator with a number derived from (a) the read name, (b) the nucleotide sequence, (c) the quality sequence, (d) the value of the `--seed` option. This means that if two reads are identical (same name, same nucleotides, same qualities) Bowtie 2 will find and report the same alignment(s) for both, even if there was ambiguity. When `--non-deterministic` is specified, Bowtie 2 re-initializes its pseudo-random generator for each read using the current time. This means that Bowtie 2 will not necessarily report the same alignment for two identical reads. This is counter-intuitive for some users, but might be more appropriate in situations where the input consists of many identical reads. |
| ``` --version ``` | Print version information and quit. |
| ``` -h/--help ``` | Print usage information and quit. |

## SAM output

Following is a brief description of the SAM format as output by `bowtie2`. For more details, see the SAM format specification.

By default, `bowtie2` prints a SAM header with `@HD`, `@SQ` and `@PG` lines. When one or more `--rg` arguments are specified, `bowtie2` will also print an `@RG` line that includes all user-specified `--rg` tokens separated by tabs.

Each subsequnt line describes an alignment or, if the read failed to align, a read. Each line is a collection of at least 12 fields separated by tabs; from left to right, the fields are:

1. Name of read that aligned.

   Note that the SAM specification disallows whitespace in the read name. If the read name contains any whitespace characters, Bowtie 2 will truncate the name at the first whitespace character. This is similar to the behavior of other tools.
2. Sum of all applicable flags. Flags relevant to Bowtie are:

   |  |  |
   | --- | --- |
   | ``` 1 ``` | The read is one of a pair |
   | ``` 2 ``` | The alignment is one end of a proper paired-end alignment |
   | ``` 4 ``` | The read has no reported alignments |
   | ``` 8 ``` | The read is one of a pair and has no reported alignments |
   | ``` 16 ``` | The alignment is to the reverse reference strand |
   | ``` 32 ``` | The other mate in the paired-end alignment is aligned to the reverse reference strand |
   | ``` 64 ``` | The read is mate 1 in a pair |
   | ``` 128 ``` | The read is mate 2 in a pair |

   Thus, an unpaired read that aligns to the reverse reference strand will have flag 16. A paired-end read that aligns and is the first mate in the pair will have flag 83 (= 64 + 16 + 2 + 1).
3. Name of reference sequence where alignment occurs
4. 1-based offset into the forward reference strand where leftmost character of the alignment occurs
5. Mapping quality
6. CIGAR string representation of alignment
7. Name of reference sequence where mate's alignment occurs. Set to `=` if the mate's reference sequence is the same as this alignment's, or `*` if there is no mate.
8. 1-based offset into the forward reference strand where leftmost character of the mate's alignment occurs. Offset is 0 if there is no mate.
9. Inferred fragment length. Size is negative if the mate's alignment occurs upstream of this alignment. Size is 0 if the mates did not align concordantly. However, size is non-0 if the mates aligned discordantly to the same chromosome.
10. Read sequence (reverse-complemented if aligned to the reverse strand)
11. ASCII-encoded read qualities (reverse-complemented if the read aligned to the reverse strand). The encoded quality values are on the Phred quality scale and the encoding is ASCII-offset by 33 (ASCII char `!`), similarly to a FASTQ file.
12. Optional fields. Fields are tab-separated. `bowtie2` outputs zero or more of these optional fields for each alignment, depending on the type of the alignment:

    |  |
    | --- |
    |  |

```
    AS:i:<N>

</td>
<td>

Alignment score.  Can be negative.  Can be greater than 0 in [`--local`]
mode (but not in [`--end-to-end`] mode).  Only present if SAM record is for
an aligned read.

</td></tr>
<tr><td id="bowtie2-build-opt-fields-xs">
```

```
    XS:i:<N>

</td>
<td>

Alignment score for second-best alignment.  Can be negative.  Can be greater
than 0 in [`--local`] mode (but not in [`--end-to-end`] mode).  Only present
if the SAM record is for an aligned read and more than one alignment was
found for the read.

</td></tr>
<tr><td id="bowtie2-build-opt-fields-ys">
```

```
    YS:i:<N>

</td>
<td>

Alignment score for opposite mate in the paired-end alignment.  Only present
if the SAM record is for a read that aligned as part of a paired-end
alignment.

</td></tr>
<tr><td id="bowtie2-build-opt-fields-xn">
```

```
    XN:i:<N>

</td>
<td>

The number of ambiguous bases in the reference covering this alignment. 
Only present if SAM record is for an aligned read.

</td></tr>
<tr><td id="bowtie2-build-opt-fields-xm">
```

```
    XM:i:<N>

</td>
<td>

The number of mismatches in the alignment.  Only present if SAM record is
for an aligned read.

</td></tr>
<tr><td id="bowtie2-build-opt-fields-xo">
```

```
    XO:i:<N>

</td>
<td>

The number of gap opens, for both read and reference gaps, in the alignment.
Only present if SAM record is for an aligned read.

</td></tr>
<tr><td id="bowtie2-build-opt-fields-xg">
```

```
    XG:i:<N>

</td>
<td>

The number of gap extensions, for both read and reference gaps, in the
alignment. Only present if SAM record is for an aligned read.

</td></tr>
<tr><td id="bowtie2-build-opt-fields-nm">
```

```
    NM:i:<N>

</td>
<td>

The edit distance; that is, the minimal number of one-nucleotide edits
(substitutions, insertions and deletions) needed to transform the read
string into the reference string.  Only present if SAM record is for an
aligned read.

</td></tr>
<tr><td id="bowtie2-build-opt-fields-yf">
```

```
    YF:Z:<S>

</td><td>

String indicating reason why the read was filtered out.  See also:
[Filtering].  Only appears for reads that were filtered out.

</td></tr>
<tr><td id="bowtie2-build-opt-fields-yt">
```

```
    YT:Z:<S>

</td><td>

Value of `UU` indicates the read was not part of a pair.  Value of `CP`
indicates the read was part of a pair and the pair aligned concordantly.
Value of `DP` indicates the read was part of a pair and the pair aligned
discordantly.  Value of `UP` indicates the read was part of a pair but the
pair failed to aligned either concordantly or discordantly.
```

```
</td></tr>
<tr><td id="bowtie2-build-opt-fields-md">
```

```
    MD:Z:<S>

</td><td>

A string representation of the mismatched reference bases in the alignment. 
See [SAM] format specification for details.  Only present if SAM record is
for an aligned read.

</td></tr>
</table>
```

# The `bowtie2-build` indexer

`bowtie2-build` builds a Bowtie index from a set of DNA sequences. `bowtie2-build` outputs a set of 6 files with suffixes `.1.bt2`, `.2.bt2`, `.3.bt2`, `.4.bt2`, `.rev.1.bt2`, and `.rev.2.bt2`. These files together constitute the index: they are all that is needed to align reads to that reference. The original sequence FASTA files are no longer used by Bowtie 2 once the index is built.

Bowtie 2's `.bt2` index format is different from Bowtie 1's `.ebwt` format, and they are not compatible with each other.

Use of Karkkainen's blockwise algorithm allows `bowtie2-build` to trade off between running time and memory usage. `bowtie2-build` has three options governing how it makes this trade: `-p`/`--packed`, `--bmax`/`--bmaxdivn`, and `--dcv`. By default, `bowtie2-build` will automatically search for the settings that yield the best running time without exhausting memory. This behavior can be disabled using the `-a`/`--noauto` option.

The indexer provides options pertaining to the "shape" of the index, e.g. `--offrate` governs the fraction of Burrows-Wheeler rows that are "marked" (i.e., the density of the suffix-array sample; see the original FM Index paper for details). All of these options are potentially profitable trade-offs depending on the application. They have been set to defaults that are reasonable for most cases according to our experiments. See Performance tuning for details.

Because `bowtie2-build` uses 32-bit pointers internally, it can handle up to a theoretical maximum of 2^32-1 (somewhat more than 4 billion) characters in an index, though, with other constraints, the actual ceiling is somewhat less than that. If your reference exceeds 2^32-1 characters, `bowtie2-build` will print an error message and abort. To resolve this, divide your reference sequences into smaller batches and/or chunks and build a separate index for each.

If your computer has more than 3-4 GB of memory and you would like to exploit that fact to make index building faster, use a 64-bit version of the `bowtie2-build` binary. The 32-bit version of the binary is restricted to using less than 4 GB of memory. If a 64-bit pre-built binary does not yet exist for your platform on the sourceforge download site, you will need to build one from source.

The Bowtie 2 index is based on the FM Index of Ferragina and Manzini, which in turn is based on the Burrows-Wheeler transform. The algorithm used to build the index is based on the blockwise algorithm of Karkkainen.

## Command Line

Usage:

```
bowtie2-build [options]* <reference_in> <bt2_base>
```

### Main arguments

|  |  |
| --- | --- |
| ``` <reference_in> ``` | A comma-separated list of FASTA files containing the reference sequences to be aligned to, or, if `-c` is specified, the sequences themselves. E.g., `<reference_in>` might be `chr1.fa,chr2.fa,chrX.fa,chrY.fa`, or, if `-c` is specified, this might be `GGTCATCCT,ACGGGTCGT,CCGTTCTATGCGGCTTA`. |
| ``` <bt2_base> ``` | The basename of the index files to write. By default, `bowtie2-build` writes files named `NAME.1.bt2`, `NAME.2.bt2`, `NAME.3.bt2`, `NAME.4.bt2`, `NAME.rev.1.bt2`, and `NAME.rev.2.bt2`, where `NAME` is `<bt2_base>`. |

### Options

|  |  |
| --- | --- |
| ``` -f ``` | The reference input files (specified as `<reference_in>`) are FASTA files (usually having extension `.fa`, `.mfa`, `.fna` or similar). |
| ``` -c ``` | The reference sequences are given on the command line. I.e. `<reference_in>` is a comma-separated list of sequences rather than a list of FASTA files. |
| ``` -a/--noauto ``` | Disable the default behavior whereby `bowtie2-build` automatically selects values for the `--bmax`, `--dcv` and `--packed` parameters according to available memory. Instead, user may specify values for those parameters. If memory is exhausted during indexing, an error message will be printed; it is up to the user to try new parameters. |
| ``` -p/--packed ``` | Use a packed (2-bits-per-nucleotide) representation for DNA strings. This saves memory but makes indexing 2-3 times slower. Default: off. This is configured automatically by default; use `-a`/`--noauto` to configure manually. |
| ``` --bmax <int> ``` | The maximum number of suffixes allowed in a block. Allowing more suffixes per block makes indexing faster, but increases peak memory usage. Setting this option overrides any previous setting for `--bmax`, or `--bmaxdivn`. Default (in terms of the `--bmaxdivn` parameter) is `--bmaxdivn` 4. This is configured automatically by default; use `-a`/`--noauto` to configure manually. |
| ``` --bmaxdivn <int> ``` | The maximum number of suffixes allowed in a block, expressed as a fraction of the length of the reference. Setting this option overrides any previous setting for `--bmax`, or `--bmaxdivn`. Default: `--bmaxdivn` 4. This is configured automatically by default; use `-a`/`--noauto` to configure manually. |
| ``` --dcv <int> ``` | Use `<int>` as the period for the difference-cover sample. A larger period yields less memory overhead, but may make suffix sorting slower, especially if repeats are present. Must be a power of 2 no greater than 4096. Default: 1024. This is configured automatically by default; use `-a`/`--noauto` to configure manually. |
| ``` --nodc ``` | Disable use of the difference-cover sample. Suffix sorting becomes quadratic-time in the worst case (where the worst case is an extremely repetitive reference). Default: off. |
| ``` -r/--noref ``` | Do not build the `NAME.3.bt2` and `NAME.4.bt2` portions of the index, which contain a bitpacked version of the reference sequences and are used for paired-end alignment. |
| ``` -3/--justref ``` | Build only the `NAME.3.bt2` and `NAME.4.bt2` portions of the index, which contain a bitpacked version of the reference sequences and are used for paired-end alignment. |
| ``` -o/--offrate <int> ``` | To map alignments back to positions on the reference sequences, it's necessary to annotate ("mark") some or all of the Burrows-Wheeler rows with their corresponding location on the genome. `-o`/`--offrate` governs how many rows get marked: the indexer will mark every 2^`<int>` rows. Marking more rows makes reference-position lookups faster, but requires more memory to hold the annotations at runtime. The default is 5 (every 32nd row is marked; for human genome, annotations occupy about 340 megabytes). |
| ``` -t/--ftabchars <int> ``` | The ftab is the lookup table used to calculate an initial Burrows-Wheeler range with respect to the first `<int>` characters of the query. A larger `<int>` yields a larger lookup table but faster query times. The ftab has size 4^(`<int>`+1) bytes. The default setting is 10 (ftab is 4MB). |
| ``` --seed <int> ``` | Use `<int>` as the seed for pseudo-random number generator. |
| ``` --cutoff <int> ``` | Index only the first `<int>` bases of the reference sequences (cumulative across sequences) and ignore the rest. |
| ``` -q/--quiet ``` | `bowtie2-build` is verbose by default. With this option `bowtie2-build` will print only error messages. |
| ``` -h/--help ``` | Print usage information and quit. |
| ``` --version ``` | Print version information and quit. |

# The `bowtie2-inspect` index inspector

`bowtie2-inspect` extracts information from a Bowtie index about what kind of index it is and what reference sequences were used to build it. When run without any options, the tool will output a FASTA file containing the sequences of the original references (with all non-`A`/`C`/`G`/`T` characters converted to `N`s). It can also be used to extract just the reference sequence names using the `-n`/`--names` option or a more verbose summary using the `-s`/`--summary` option.

## Command Line

Usage:

```
bowtie2-inspect [options]* <bt2_base>
```

### Main arguments

|  |  |
| --- | --- |
| ``` <bt2_base> ``` | The basename of the index to be inspected. The basename is name of any of the index files but with the `.X.bt2` or `.rev.X.bt2` suffix omitted. `bowtie2-inspect` first looks in the current directory for the index files, then in the directory specified in the `BOWTIE2_INDEXES` environment variable. |

### Options

|  |  |
| --- | --- |
| ``` -a/--across <int> ``` | When printing FASTA output, output a newline character every `<int>` bases (default: 60). |
| ``` -n/--names ``` | Print reference sequence names, one per line, and quit. |
| ``` -s/--summary ``` | Print a summary that includes information about index settings, as well as the names and lengths of the input sequences. The summary has this format:   ``` Colorspace  <0 or 1> SA-Sample   1 in <sample> FTab-Chars  <chars> Sequence-1  <name>  <len> Sequence-2  <name>  <len> ... Sequence-N  <name>  <len> ```   Fields are separated by tabs. Colorspace is always set to 0 for Bowtie 2. |
| ``` -v/--verbose ``` | Print verbose output (for debugging). |
| ``` --version ``` | Print version information and quit. |
| ``` -h/--help ``` | Print usage information and quit. |

# Getting started with Bowtie 2: Lambda phage example

Bowtie 2 comes with some example files to get you started. The example files are not scientifically significant; we use the Lambda phage reference genome simply because it's short, and the reads were generated by a computer program, not a sequencer. However, these files will let you start running Bowtie 2 and downstream tools right away.

First follow the manual instructions to obtain Bowtie 2. Set the `BT2_HOME` environment variable to point to the new Bowtie 2 directory containing the `bowtie2`, `bowtie2-build` and `bowtie2-inspect` binaries. This is important, as the `BT2_HOME` variable is used in the commands below to refer to that directory.

## Indexing a reference genome

To create an index for the Lambda phage reference genome included with Bowtie 2, create a new temporary directory (it doesn't matter where), change into that directory, and run:

```
$BT2_HOME/bowtie2-build $BT2_HOME/example/reference/lambda_virus.fa lambda_virus
```

The command should print many lines of output then quit. When the command completes, the current directory will contain four new files that all start with `lambda_virus` and end with `.1.bt2`, `.2.bt2`, `.3.bt2`, `.4.bt2`, `.rev.1.bt2`, and `.rev.2.bt2`. These files constitute the index - you're done!

You can use `bowtie2-build` to create an index for a set of FASTA files obtained from any source, including sites such as UCSC, NCBI, and Ensembl. When indexing multiple FASTA files, specify all the files using commas to separate file names. For more details on how to create an index with `bowtie2-build`, see the manual section on index building. You may also want to bypass this process by obtaining a pre-built index. See using a pre-built index below for an example.

## Aligning example reads

Stay in the directory created in the previous step, which now contains the `lambda_virus` index files. Next, run:

```
$BT2_HOME/bowtie2 -x lambda_virus -U $BT2_HOME/example/reads/reads_1.fq -S eg1.sam
```

This runs the Bowtie 2 aligner, which aligns a set of unpaired reads to the Lambda phage reference genome using the index generated in the previous step. The alignment results in SAM format are written to the file `eg1.sam`, and a short alignment summary is written to the console. (Actually, the summary is written to the "standard error" or "stderr" filehandle, which is typically printed to the console.)

To see the first few lines of the SAM output, run:

```
head eg1.sam
```

You will see something like this:

```
@HD VN:1.0  SO:unsorted
@SQ SN:gi|9626243|ref|NC_001416.1|  LN:48502
@PG ID:bowtie2  PN:bowtie2  VN:2.0.1
r1  0   gi|9626243|ref|NC_001416.1| 18401   42  122M    *   0   0   TGAATGCGAACTCCGGGACGCTCAGTAATGTGACGATAGCTGAAAACTGTACGATAAACNGTACGCTGAGGGCAGAAAAAATCGTCGGGGACATTNTAAAGGCGGCGAGCGCGGCTTTTCCG  +"@6<:27(F&5)9)"B:%B+A-%5A?2$HCB0B+0=D<7E/<.03#!.F77@6B==?C"7>;))%;,3-$.A06+<-1/@@?,26">=?*@'0;$:;??G+:#+(A?9+10!8!?()?7C>  AS:i:-5 XN:i:0  XM:i:3  XO:i:0  XG:i:0  NM:i:3  MD:Z:59G13G21G26    YT:Z:UU
r2  0   gi|9626243|ref|NC_001416.1| 8886    42  275M    *   0   0   NTTNTGATGCGGGCTTGTGGAGTTCAGCCGATCTGACTTATGTCATTACCTATGAAATGTGAGGACGCTATGCCTGTACCAAATCCTACAATGCCGGTGAAAGGTGCCGGGATCACCCTGTGGGTTTATAAGGGGATCGGTGACCCCTACGCGAATCCGCTTTCAGACGTTGACTGGTCGCGTCTGGCAAAAGTTAAAGACCTGACGCCCGGCGAACTGACCGCTGAGNCCTATGACGACAGCTATCTCGATGATGAAGATGCAGACTGGACTGC (#!!'+!$""%+(+)'%)%!+!(&++)''"#"#&#"!'!("%'""("+&%$%*%%#$%#%#!)*'(#")(($&$'&%+&#%*)*#*%*')(%+!%%*"$%"#+)$&&+)&)*+!"*)!*!("&&"*#+"&"'(%)*("'!$*!!%$&&&$!!&&"(*"$&"#&!$%'%"#)$#+%*+)!&*)+(""#!)!%*#"*)*')&")($+*%%)!*)!('(%""+%"$##"#+(('!*(($*'!"*('"+)&%#&$+('**$$&+*&!#%)')'(+(!%+ AS:i:-14    XN:i:0  XM:i:8  XO:i:0  XG:i:0  NM:i:8  MD:Z:0A0C0G0A108C23G9T81T46 YT:Z:UU
r3  16  gi|9626243|ref|NC_001416.1| 11599   42  338M    *   0   0   GGGCGCGTTACTGGGATGATCGTGAAAAGGCCCGTCTTGCGCTTGAAGCCGCCCGAAAGAAGGCTGAGCAGCAGACTCAAGAGGAGAAAAATGCGCAGCAGCGGAGCGATACCGAAGCGTCACGGCTGAAATATACCGAAGAGGCGCAGAAGGCTNACGAACGGCTGCAGACGCCGCTGCAGAAATATACCGCCCGTCAGGAAGAACTGANCAAGGCACNGAAAGACGGGAAAATCCTGCAGGCGGATTACAACACGCTGATGGCGGCGGCGAAAAAGGATTATGAAGCGACGCTGTAAAAGCCGAAACAGTCCAGCGTGAAGGTGTCTGCGGGCGAT  7F$%6=$:9B@/F'>=?!D?@0(:A*)7/>9C>6#1<6:C(.CC;#.;>;2'$4D:?&B!>689?(0(G7+0=@37F)GG=>?958.D2E04C<E,*AD%G0.%$+A:'H;?8<72:88?E6((CF)6DF#.)=>B>D-="C'B080E'5BH"77':"@70#4%A5=6.2/1>;9"&-H6)=$/0;5E:<8G!@::1?2DC7C*;@*#.1C0.D>H/20,!"C-#,6@%<+<D(AG-).?&#0.00'@)/F8?B!&"170,)>:?<A7#1(A@0E#&A.*DC.E")AH"+.,5,2>5"2?:G,F"D0B8D-6$65D<D!A/38860.*4;4B<*31?6  AS:i:-22    XN:i:0  XM:i:8  XO:i:0  XG:i:0  NM:i:8  MD:Z:80C4C16A52T23G30A8T76A41   YT:Z:UU
r4  0   gi|9626243|ref|NC_001416.1| 40075   42  184M    *   0   0   GGGCCAATGCGCTTACTGATGCGGAATTACGCCGTAAGGCCGCAGATGAGCTTGTCCATATGACTGCGAGAATTAACNGTGGTGAGGCGATCCCTGAACCAGTAAAACAACTTCCTGTCATGGGCGGTAGACCTCTAAATCGTGCACAGGCTCTGGCGAAGATCGCAGAAATCAAAGCTAAGT(=8B)GD04*G%&4F,1'A>.C&7=F$,+#6!))43C,5/5+)?-/0>/D3=-,2/+.1?@->;)00!'3!7BH$G)HG+ADC'#-9F)7<7"$?&.>0)@5;4,!0-#C!15CF8&HB+B==H>7,/)C5)5*+(F5A%D,EA<(>G9E0>7&/E?4%;#'92)<5+@7:A.(BG@BG86@.G AS:i:-1 XN:i:0  XM:i:1  XO:i:0  XG:i:0  NM:i:1  MD:Z:77C106 YT:Z:UU
r5  0   gi|9626243|ref|NC_001416.1| 48010   42  138M    *   0   0   GTCAGGAAAGTGGTAAAACTGCAACTCAATTACTGCAATGCCCTCGTAATTAAGTGAATTTACAATATCGTCCTGTTCGGAGGGAAGAACGCGGGATGTTCATTCTTCATCACTTTTAATTGATGTATATGCTCTCTT  9''%<D)A03E1-*7=),:F/0!6,D9:H,<9D%:0B(%'E,(8EFG$E89B$27G8F*2+4,-!,0D5()&=(FGG:5;3*@/.0F-G#5#3->('FDFEG?)5.!)"AGADB3?6(@H(:B<>6!>;>6>G,."?%  AS:i:0  XN:i:0  XM:i:0  XO:i:0  XG:i:0  NM:i:0  MD:Z:138    YT:Z:UU
r6  16  gi|9626243|ref|NC_001416.1| 41607   42  72M2D119M   *   0   0   TCGATTTGCAAATACCGGAACATCTCGGTAACTGCATATTCTGCATTAAAAAATCAACGCAAAAAATCGGACGCCTGCAAAGATGAGGAGGGATTGCAGCGTGTTTTTAATGAGGTCATCACGGGATNCCATGTGCGTGACGGNCATCGGGAAACGCCAAAGGAGATTATGTACCGAGGAAGAATGTCGCT 1H#G;H"$E*E#&"*)2%66?=9/9'=;4)4/>@%+5#@#$4A*!<D=="8#1*A9BA=:(1+#C&.#(3#H=9E)AC*5,AC#E'536*2?)H14?>9'B=7(3H/B:+A:8%1-+#(E%&$$&14"76D?>7(&20H5%*&CF8!G5B+A4F$7(:"'?0$?G+$)B-?2<0<F=D!38BH,%=8&5@+ AS:i:-13    XN:i:0  XM:i:2  XO:i:1  XG:i:2  NM:i:4  MD:Z:72^TT55C15A47  YT:Z:UU
r7  16  gi|9626243|ref|NC_001416.1| 4692    42  143M    *   0   0   TCAGCCGGACGCGGGCGCTGCAGCCGTACTCGGGGATGACCGGTTACAACGGCATTATCGCCCGTCTGCAACAGGCTGCCAGCGATCCGATGGTGGACAGCATTCTGCTCGATATGGACANGCCCGGCGGGATGGTGGCGGGG -"/@*7A0)>2,AAH@&"%B)*5*23B/,)90.B@%=FE,E063C9?,:26$-0:,.,1849'4.;F>FA;76+5&$<C":$!A*,<B,<)@<'85D%C*:)30@85;?.B$05=@95DCDH<53!8G:F:B7/A.E':434> AS:i:-6 XN:i:0  XM:i:2  XO:i:0  XG:i:0  NM:i:2  MD:Z:98G21C22   YT:Z:UU
```

The first few lines (beginning with `@`) are SAM header lines, and the rest of the lines are SAM alignments, one line per read or mate. See the Bowtie 2 manual section on SAM output and the SAM specification for details about how to interpret the SAM file format.

## Paired-end example

To align paired-end reads included with Bowtie 2, stay in the same directory and run:

```
$BT2_HOME/bowtie2 -x lambda_virus -1 $BT2_HOME/example/reads/reads_1.fq -2 $BT2_HOME/example/reads/reads_2.fq -S eg2.sam
```

This aligns a set of paired-end reads to the reference genome, with results written to the file `eg2.sam`.

## Local alignment example

To use local alignment to align some longer reads included with Bowtie 2, stay in the same directory and run:

```
$BT2_HOME/bowtie2 --local -x lambda_virus -U $BT2_HOME/example/reads/longreads.fq -S eg3.sam
```

This aligns the long reads to the reference genome using local alignment, with results written to the file `eg3.sam`.

## Using SAMtools/BCFtools downstream

SAMtools is a collection of tools for manipulating and analyzing SAM and BAM alignment files. BCFtools is a collection of tools for calling variants and manipulating VCF and BCF files, and it is typically distributed with SAMtools. Using these tools together allows you to get from alignments in SAM format to variant calls in VCF format. This example assumes that `samtools` and `bcftools` are installed and that the directories containing these binaries are in your PATH environment variable.

Run the paired-end example:

```
$BT2_HOME/bowtie2 -x $BT2_HOME/example/index/lambda_virus -1 $BT2_HOME/example/reads/reads_1.fq -2 $BT2_HOME/example/reads/reads_2.fq -S eg2.sam
```

Use `samtools view` to convert the SAM file into a BAM file. BAM is a the binary format corresponding to the SAM text format. Run:

```
samtools view -bS eg2.sam > eg2.bam
```

Use `samtools sort` to convert the BAM file to a sorted BAM file.

```
samtools sort eg2.bam eg2.sorted
```

We now have a sorted BAM file called `eg2.sorted.bam`. Sorted BAM is a useful format because the alignments are (a) compressed, which is convenient for long-term storage, and (b) sorted, which is conveneint for variant discovery. To generate variant calls in VCF format, run:

```
samtools mpileup -uf $BT2_HOME/example/reference/lambda_virus.fa eg2.sorted.bam | bcftools view -bvcg - > eg2.raw.bcf
```

Then to view the variants, run:

```
bcftools view eg2.raw.bcf
```

See the official SAMtools guide to Calling SNPs/INDELs with SAMtools/BCFtools for more details and variations on this process.
