# Supplementary material for: Centrifuge: rapid and sensitive classification of metagenomic sequences
Source: Genome Res. 2016 Dec;26(12):1721–9. doi: 10.1101/gr.210641.116 (PMC5131823; doi:10.1101/gr.210641.116)
Supplement: Supplemental Material [file supp_gr.210641.116_Supplemental_Data_S1.zip › centrifuge-centrifuge-genome-research/doc/manual.inc.html]

- Introduction
  - What is Centrifuge?
- Obtaining Centrifuge
  - Building from source
- Running Centrifuge
  - Adding to PATH
  - Before running Centrifuge
  - Database download and index building
    - Building index on all complete bacterial and viral genomes
    - Adding human or mouse genome to the index
    - nt database
    - Custom database
    - Centrifuge classification output
    - Centrifuge summary output (the default filename is centrifuge\_report.tsv)
  - Inspecting the Centrifuge index
  - Wrapper
  - Performance tuning
  - Command Line
    - Usage
    - Main arguments
    - Options
- The `centrifuge-build` indexer
  - Command Line
    - Main arguments
    - Options
- The `centrifuge-inspect` index inspector
  - Command Line
    - Main arguments
    - Options
- Getting started with Centrifuge
  - Indexing a reference genome
  - Classifying example reads

# Introduction

## What is Centrifuge?

Centrifuge is a novel microbial classification engine that enables rapid, accurate, and sensitive labeling of reads and quantification of species on desktop computers. The system uses a novel indexing scheme based on the Burrows-Wheeler transform (BWT) and the Ferragina-Manzini (FM) index, optimized specifically for the metagenomic classification problem. Centrifuge requires a relatively small index (5.8 GB for all complete bacterial and viral genomes plus the human genome) and classifies sequences at a very high speed, allowing it to process the millions of reads from a typical high-throughput DNA sequencing run within a few minutes. Together these advances enable timely and accurate analysis of large metagenomics data sets on conventional desktop computers.

# Obtaining Centrifuge

Download Centrifuge and binaries from the Releases sections on the right side. Binaries are available for Intel architectures (`x86_64`) running Linux, and Mac OS X.

## Building from source

Building Centrifuge from source requires a GNU-like environment with GCC, GNU Make and other basics. It should be possible to build Centrifuge on most vanilla Linux installations or on a Mac installation with Xcode installed. Centrifuge can also be built on Windows using Cygwin or MinGW (MinGW recommended). For a MinGW build the choice of what compiler is to be used is important since this will determine if a 32 or 64 bit code can be successfully compiled using it. If there is a need to generate both 32 and 64 bit on the same machine then a multilib MinGW has to be properly installed. MSYS, the zlib library, and depending on architecture pthreads library are also required. We are recommending a 64 bit build since it has some clear advantages in real life research problems. In order to simplify the MinGW setup it might be worth investigating popular MinGW personal builds since these are coming already prepared with most of the toolchains needed.

First, download the [source package] from the Releases secion on the right side. Unzip the file, change to the unzipped directory, and build the Centrifuge tools by running GNU `make` (usually with the command `make`, but sometimes with `gmake`) with no arguments. If building with MinGW, run `make` from the MSYS environment.

Centrifuge is using the multithreading software model in order to speed up execution times on SMP architectures where this is possible. On POSIX platforms (like linux, Mac OS, etc) it needs the pthread library. Although it is possible to use pthread library on non-POSIX platform like Windows, due to performance reasons Centrifuge will try to use Windows native multithreading if possible.

For the support of SRA data access in HISAT2, please download and install the NCBI-NGS toolkit. When running `make`, specify additional variables as follow. `make USE_SRA=1 NCBI_NGS_DIR=/path/to/NCBI-NGS-directory NCBI_VDB_DIR=/path/to/NCBI-NGS-directory`, where `NCBI_NGS_DIR` and `NCBI_VDB_DIR` will be used in Makefile for -I and -L compilation options. For example, $(NCBI\_NGS\_DIR)/include and $(NCBI\_NGS\_DIR)/lib64 will be used.

# Running Centrifuge

## Adding to PATH

By adding your new Centrifuge directory to your PATH environment variable, you ensure that whenever you run `centrifuge`, `centrifuge-build`, `centrifuge-download` or `centrifuge-inspect` from the command line, you will get the version you just installed without having to specify the entire path. This is recommended for most users. To do this, follow your operating system's instructions for adding the directory to your PATH.

If you would like to install Centrifuge by copying the Centrifuge executable files to an existing directory in your PATH, make sure that you copy all the executables, including `centrifuge`, `centrifuge-class`, `centrifuge-build`, `centrifuge-build-bin`, `centrifuge-download` `centrifuge-inspect` and `centrifuge-inspect-bin`. Furthermore you need the programs in the scripts/ folder if you opt for genome compression in the database construction.

## Before running Centrifuge

Classification is considerably different from alignment in that classification is performed on a large set of genomes as opposed to on just one reference genome as in alignment. Currently, an enormous number of complete genomes are available at the GenBank (e.g. >4,000 bacterial genomes, >10,000 viral genomes, …). These genomes are organized in a taxonomic tree where each genome is located at the bottom of the tree, at the strain or subspecies level. On the taxonomic tree, genomes have ancestors usually situated at the species level, and those ancestors also have ancestors at the genus level and so on up the family level, the order level, class level, phylum, kingdom, and finally at the root level.

Given the gigantic number of genomes available, which continues to expand at a rapid rate, and the development of the taxonomic tree, which continues to evolve with new advancements in research, we have designed Centrifuge to be flexible and general enough to reflect this huge database. We provide several standard indexes that will meet most of users’ needs (see the side panel - Indexes). In our approach our indexes not only include raw genome sequences, but also genome names/sizes and taxonomic trees. This enables users to perform additional analyses on Centrifuge’s classification output without the need to download extra database sources. This also eliminates the potential issue of discrepancy between the indexes we provide and the databases users may otherwise download. We plan to provide a couple of additional standard indexes in the near future, and update the indexes on a regular basis.

We encourage first time users to take a look at and follow a `small example` that illustrates how to build an index, how to run Centrifuge using the index, how to interpret the classification results, and how to extract additional genomic information from the index. For those who choose to build customized indexes, please take a close look at the following description.

## Database download and index building

Centrifuge indexes can be built with arbritary sequences. Standard choices are all of the complete bacterial and viral genomes, or using the sequences that are part of the BLAST nt database. Centrifuge always needs the nodes.dmp file from the NCBI taxonomy dump to build the taxonomy tree, as well as a sequence ID to taxonomy ID map. The map is a tab-separated file with the sequence ID to taxonomy ID map.

To download all of the complete archaeal, viral, and bacterial genomes from RefSeq, and build the index:

Centrifuge indices can be build on arbritary sequences. Usually an ensemble of genomes is used - such as all complete microbial genomes in the RefSeq database, or all sequences in the BLAST nt database.

To map sequence identifiers to taxonomy IDs, and taxonomy IDs to names and its parents, three files are necessary in addition to the sequence files:

- taxonomy tree: typically nodes.dmp from the NCBI taxonomy dump. Links taxonomy IDs to their parents
- names file: typically names.dmp from the NCBI taxonomy dump. Links taxonomy IDs to their scientific name
- a tab-separated sequence ID to taxonomy ID mapping

When using the provided scripts to download the genomes, these files are automatically downloaded or generated. When using a custom taxonomy or sequence files, please refer to the section `TODO` to learn more about their format.

### Building index on all complete bacterial and viral genomes

Use `centrifuge-download` to download genomes from NCBI. The following two commands download the NCBI taxonomy to `taxonomy/` in the current directory, and all complete archaeal, bacterial and viral genomes to `library/`. Low-complexity regions in the genomes are masked after download (parameter `-m`) using blast+'s `dustmasker`. `centrifuge-download` outputs tab-separated sequence ID to taxonomy ID mappings to standard out, which are required by `centrifuge-build`.

```
centrifuge-download -o taxonomy taxonomy
centrifuge-download -o library -m -d "archaea,bacteria,viral" refseq > seqid2taxid.map
```

To build the index, first concatenate all downloaded sequences into a single file, and then run `centrifuge-build`:

```
cat library/*/*.fna > input-sequences.fna

## build centrifuge index with 4 threads
centrifuge-build -p 4 --conversion-table seqid2taxid.map \
                 --taxonomy-tree taxonomy/nodes.dmp --name-table taxonomy/names.dmp \
                 input-sequences.fna abv
```

After building the index, all files except the index \*.[123].cf files may be removed. If you also want to include the human and/or the mouse genome, add their sequences to the library folder before building the index with one of the following commands:

After the index building, all but the \*.[123].cf index files may be removed. I.e. the files in the `library/` and `taxonomy/` directories are no longer needed.

### Adding human or mouse genome to the index

The human and mouse genomes can also be downloaded using `centrifuge-download`. They are in the domain "vertebrate\_mammalian" (argument `-d`), are assembled at the chromosome level (argument `-a`) and categorized as reference genomes by RefSeq (`-c`). The argument `-t` takes a comma-separated list of taxonomy IDs - e.g. `9606` for human and `10090` for mouse:

```
# download mouse and human reference genomes
centrifuge-download -o library -d "vertebrate_mammalian" -a "Chromosome" -t 9606,10090 -c 'reference genome' >> seqid2taxid.map
# only human
centrifuge-download -o library -d "vertebrate_mammalian" -a "Chromosome" -t 9606 -c 'reference genome' >> seqid2taxid.map
# only mouse
centrifuge-download -o library -d "vertebrate_mammalian" -a "Chromosome" -t 10090 -c 'reference genome' >> seqid2taxid.map
```

### nt database

NCBI BLAST's nt database contains all spliced non-redundant coding sequences from multiplpe databases, inferred from genommic sequences. Traditionally used with BLAST, a download of the FASTA is provided on the NCBI homepage. Building an index with any database requires the user to creates a sequence ID to taxonomy ID map that can be generated from a GI taxid dump:

```
wget ftp://ftp.ncbi.nih.gov/blast/db/FASTA/nt.gz
gunzip nt.gz && mv -v nt nt.fa

# Get mapping file
wget ftp://ftp.ncbi.nih.gov/pub/taxonomy/gi_taxid_nucl.dmp.gz
gunzip -c gi_taxid_nucl.dmp.gz | sed 's/^/gi|/' > gi_taxid_nucl.map

# build index using 16 cores and a small bucket size, which will require less memory
centrifuge-build -p 16 --bmax 1342177280 --conversion-table gi_taxid_nucl.map \
                 --taxonomy-tree taxonomy/nodes.dmp --name-table taxonomy/names.dmp \ 
                 nt.fa nt
```

### Custom database

TODO: Add toy example for nodes.dmp, names.dmp and seqid2taxid.map

### Centrifuge classification output

The following example shows classification assignments for a read. The assignment output has 8 columns.

```
readID    seqID   taxID score      2ndBestScore    hitLength    queryLength numMatches
1_1       gi|4    9646  4225       0               80   80      1

The first column is the read ID from a raw sequencing read (e.g., 1_1 in the example).
The second column is the sequence ID of the genomic sequence, where the read is classified (e.g., gi|4).
The third column is the taxonomic ID of the genomic sequence in the second column (e.g., 9646).
The fourth column is the score for the classification, which is the weighted sum of hits (e.g., 4225)
The fifth column is the score for the next best classification (e.g., 0).
The sixth column is a pair of two numbers: (1) an approximate number of base pairs of the read that match the genomic sequence and (2) the length of a read or the combined length of mate pairs (e.g., 80 / 80).
The seventh column is a pair of two numbers: (1) an approximate number of base pairs of the read that match the genomic sequence and (2) the length of a read or the combined length of mate pairs (e.g., 80 / 80). 
The eighth column is the number of classifications, indicating how many assignments were made (e.g.,1).
```

### Centrifuge summary output (the default filename is centrifuge\_report.tsv)

The following example shows a classification summary for each genome or taxonomic unit. The assignment output has 7 columns.

```
name                                                            taxID   taxRank    genomeSize   numReads   numUniqueReads   abundance
Wigglesworthia glossinidia endosymbiont of Glossina brevipalpis 36870   leaf       703004       5981       5964             0.0152317

The first column is the name of a genome, or the name corresponding to a taxonomic ID (the second column) at a rank higher than the strain (e.g., Wigglesworthia glossinidia endosymbiont of Glossina brevipalpis).
The second column is the taxonomic ID (e.g., 36870).
The third column is the taxonomic rank (e.g., leaf).
The fourth column is the length of the genome sequence (e.g., 703004).
The fifth column is the number of reads classified to this genomic sequence including multi-classified reads (e.g., 5981).
The sixth column is the number of reads uniquely classified to this genomic sequence (e.g., 5964).
The seventh column is the proportion of this genome normalized by its genomic length (e.g., 0.0152317).
```

## Inspecting the Centrifuge index

The index can be inspected with `centrifuge-inspect`. To extract raw sequences:

```
centrifuge-inspect <centrifuge index>
```

Extract the sequence ID to taxonomy ID conversion table from the index

```
centrifuge-inspect --conversion-table <centrifuge index>
```

Extract the taxonomy tree from the index:

```
centrifuge-inspect --taxonomy-tree <centrifuge index>
```

Extract the lengths of the sequences from the index (each row has two columns: taxonomic ID and length):

```
centrifuge-inspect --size-table <centrifuge index>
```

Extract the names from the index (each row has two columns: taxonomic ID and name):

```
centrifuge-inspect --name-table <centrifuge index>
```

## Wrapper

The `centrifuge`, `centrifuge-build` and `centrifuge-inspect` executables are actually wrapper scripts that call binary programs as appropriate. Also, the `centrifuge` wrapper provides some key functionality, like the ability to handle compressed inputs, and the functionality for [`--un`], [`--al`] and related options.

It is recommended that you always run the centrifuge wrappers and not run the binaries directly.

## Performance tuning

1. If your computer has multiple processors/cores, use `-p NTHREADS`

   The `-p` option causes Centrifuge to launch a specified number of parallel search threads. Each thread runs on a different processor/core and all threads find alignments in parallel, increasing alignment throughput by approximately a multiple of the number of threads (though in practice, speedup is somewhat worse than linear).

## Command Line

### Usage

```
centrifuge [options]* -x <centrifuge-idx> {-1 <m1> -2 <m2> | -U <r> | --sra-acc <SRA accession number>} [--report-file <report file name> -S <classification output file name>]
```

### Main arguments

|  |  |
| --- | --- |
| ``` -x <centrifuge-idx> ``` | The basename of the index for the reference genomes. The basename is the name of any of the index files up to but not including the final `.1.cf` / etc. `centrifuge` looks for the specified index first in the current directory, then in the directory specified in the `CENTRIFUGE_INDEXES` environment variable. |
| ``` -1 <m1> ``` | Comma-separated list of files containing mate 1s (filename usually includes `_1`), e.g. `-1 flyA_1.fq,flyB_1.fq`. Sequences specified with this option must correspond file-for-file and read-for-read with those specified in `<m2>`. Reads may be a mix of different lengths. If `-` is specified, `centrifuge` will read the mate 1s from the "standard in" or "stdin" filehandle. |
| ``` -2 <m2> ``` | Comma-separated list of files containing mate 2s (filename usually includes `_2`), e.g. `-2 flyA_2.fq,flyB_2.fq`. Sequences specified with this option must correspond file-for-file and read-for-read with those specified in `<m1>`. Reads may be a mix of different lengths. If `-` is specified, `centrifuge` will read the mate 2s from the "standard in" or "stdin" filehandle. |
| ``` -U <r> ``` | Comma-separated list of files containing unpaired reads to be aligned, e.g. `lane1.fq,lane2.fq,lane3.fq,lane4.fq`. Reads may be a mix of different lengths. If `-` is specified, `centrifuge` gets the reads from the "standard in" or "stdin" filehandle. |
| ``` --sra-acc <SRA accession number> ``` | Comma-separated list of SRA accession numbers, e.g. `--sra-acc SRR353653,SRR353654`. Information about read types is available at http://trace.ncbi.nlm.nih.gov/Traces/sra/sra.cgi?sp=runinfo&acc=**sra-acc**&retmode=xml, where **sra-acc** is SRA accession number. If users run HISAT2 on a computer cluster, it is recommended to disable SRA-related caching (see the instruction at SRA-MANUAL). |
| ``` -S <filename> ``` | File to write classification results to. By default, assignments are written to the "standard out" or "stdout" filehandle (i.e. the console). |
| ``` --report-file <filename> ``` | File to write a classification summary to (default: centrifuge\_report.tsv). |

### Options

#### Input options

|  |  |
| --- | --- |
| ``` -q ``` | Reads (specified with `<m1>`, `<m2>`, `<s>`) are FASTQ files. FASTQ files usually have extension `.fq` or `.fastq`. FASTQ is the default format. See also: `--solexa-quals` and `--int-quals`. |
| ``` --qseq ``` | Reads (specified with `<m1>`, `<m2>`, `<s>`) are QSEQ files. QSEQ files usually end in `_qseq.txt`. See also: `--solexa-quals` and `--int-quals`. |
| ``` -f ``` | Reads (specified with `<m1>`, `<m2>`, `<s>`) are FASTA files. FASTA files usually have extension `.fa`, `.fasta`, `.mfa`, `.fna` or similar. FASTA files do not have a way of specifying quality values, so when `-f` is set, the result is as if `--ignore-quals` is also set. |
| ``` -r ``` | Reads (specified with `<m1>`, `<m2>`, `<s>`) are files with one input sequence per line, without any other information (no read names, no qualities). When `-r` is set, the result is as if `--ignore-quals` is also set. |
| ``` -c ``` | The read sequences are given on command line. I.e. `<m1>`, `<m2>` and `<singles>` are comma-separated lists of reads rather than lists of read files. There is no way to specify read names or qualities, so `-c` also implies `--ignore-quals`. |
| ``` -s/--skip <int> ``` | Skip (i.e. do not align) the first `<int>` reads or pairs in the input. |
| ``` -u/--qupto <int> ``` | Align the first `<int>` reads or read pairs from the input (after the `-s`/`--skip` reads or pairs have been skipped), then stop. Default: no limit. |
| ``` -5/--trim5 <int> ``` | Trim `<int>` bases from 5' (left) end of each read before alignment (default: 0). |
| ``` -3/--trim3 <int> ``` | Trim `<int>` bases from 3' (right) end of each read before alignment (default: 0). |
| ``` --phred33 ``` | Input qualities are ASCII chars equal to the Phred quality plus 33. This is also called the "Phred+33" encoding, which is used by the very latest Illumina pipelines. |
| ``` --phred64 ``` | Input qualities are ASCII chars equal to the Phred quality plus 64. This is also called the "Phred+64" encoding. |
| ``` --solexa-quals ``` | Convert input qualities from Solexa (which can be negative) to Phred (which can't). This scheme was used in older Illumina GA Pipeline versions (prior to 1.3). Default: off. |
| ``` --int-quals ``` | Quality values are represented in the read input file as space-separated ASCII integers, e.g., `40 40 30 40`..., rather than ASCII characters, e.g., `II?I`.... Integers are treated as being on the Phred quality scale unless `--solexa-quals` is also specified. Default: off. |

#### Classification

|  |  |
| --- | --- |
| ``` --min-hitlen <int> ``` | Minimum length of partial hits, which must be greater than 15 (default: 22)" |
| ``` -k <int> ``` | It searches for at most `<int>` distinct, primary assignments for each read or pair. Primary assignments mean assignments whose assignment score is equal or higher than any other assignments. If there are more primary assignments than this value, the search will merge some of the assignments into a higher taxonomic rank. The assignment score for a paired-end assignment equals the sum of the assignment scores of the individual mates. Default: 5 |
| ``` --host-taxids ``` | A comma-separated list of taxonomic IDs that will be preferred in classification procedure. The descendants from these IDs will also be preferred. In case some of a read's assignments correspond to these taxonomic IDs, only those corresponding assignments will be reported. |
| ``` --exclude-taxids ``` | A comma-separated list of taxonomic IDs that will be excluded in classification procedure. The descendants from these IDs will also be exclude. |

#### Output options

|  |  |
| --- | --- |
| ``` -t/--time ``` | Print the wall-clock time required to load the index files and align the reads. This is printed to the "standard error" ("stderr") filehandle. Default: off. |
| ``` --quiet ``` | Print nothing besides alignments and serious errors. |
| ``` --met-file <path> ``` | Write `centrifuge` metrics to file `<path>`. Having alignment metric can be useful for debugging certain problems, especially performance issues. See also: `--met`. Default: metrics disabled. |
| ``` --met-stderr ``` | Write `centrifuge` metrics to the "standard error" ("stderr") filehandle. This is not mutually exclusive with `--met-file`. Having alignment metric can be useful for debugging certain problems, especially performance issues. See also: `--met`. Default: metrics disabled. |
| ``` --met <int> ``` | Write a new `centrifuge` metrics record every `<int>` seconds. Only matters if either `--met-stderr` or `--met-file` are specified. Default: 1. |

#### Performance options

|  |  |
| --- | --- |
| ``` -o/--offrate <int> ``` | Override the offrate of the index with `<int>`. If `<int>` is greater than the offrate used to build the index, then some row markings are discarded when the index is read into memory. This reduces the memory footprint of the aligner but requires more time to calculate text offsets. `<int>` must be greater than the value used to build the index. |
| ``` -p/--threads NTHREADS ``` | Launch `NTHREADS` parallel search threads (default: 1). Threads will run on separate processors/cores and synchronize when parsing reads and outputting alignments. Searching for alignments is highly parallel, and speedup is close to linear. Increasing `-p` increases Centrifuge's memory footprint. E.g. when aligning to a human genome index, increasing `-p` from 1 to 8 increases the memory footprint by a few hundred megabytes. This option is only available if `bowtie` is linked with the `pthreads` library (i.e. if `BOWTIE_PTHREADS=0` is not specified at build time). |
| ``` --reorder ``` | Guarantees that output records are printed in an order corresponding to the order of the reads in the original input file, even when `-p` is set greater than 1. Specifying `--reorder` and setting `-p` greater than 1 causes Centrifuge to run somewhat slower and use somewhat more memory then if `--reorder` were not specified. Has no effect if `-p` is set to 1, since output order will naturally correspond to input order in that case. |
| ``` --mm ``` | Use memory-mapped I/O to load the index, rather than typical file I/O. Memory-mapping allows many concurrent `bowtie` processes on the same computer to share the same memory image of the index (i.e. you pay the memory overhead just once). This facilitates memory-efficient parallelization of `bowtie` in situations where using `-p` is not possible or not preferable. |

#### Other options

|  |  |
| --- | --- |
| ``` --qc-filter ``` | Filter out reads for which the QSEQ filter field is non-zero. Only has an effect when read format is `--qseq`. Default: off. |
| ``` --seed <int> ``` | Use `<int>` as the seed for pseudo-random number generator. Default: 0. |
| ``` --non-deterministic ``` | Normally, Centrifuge re-initializes its pseudo-random generator for each read. It seeds the generator with a number derived from (a) the read name, (b) the nucleotide sequence, (c) the quality sequence, (d) the value of the `--seed` option. This means that if two reads are identical (same name, same nucleotides, same qualities) Centrifuge will find and report the same classification(s) for both, even if there was ambiguity. When `--non-deterministic` is specified, Centrifuge re-initializes its pseudo-random generator for each read using the current time. This means that Centrifuge will not necessarily report the same classification for two identical reads. This is counter-intuitive for some users, but might be more appropriate in situations where the input consists of many identical reads. |
| ``` --version ``` | Print version information and quit. |
| ``` -h/--help ``` | Print usage information and quit. |

# The `centrifuge-build` indexer

`centrifuge-build` builds a Centrifuge index from a set of DNA sequences. `centrifuge-build` outputs a set of 6 files with suffixes `.1.cf`, `.2.cf`, and `.3.cf`. These files together constitute the index: they are all that is needed to align reads to that reference. The original sequence FASTA files are no longer used by Centrifuge once the index is built.

Use of Karkkainen's blockwise algorithm allows `centrifuge-build` to trade off between running time and memory usage. `centrifuge-build` has two options governing how it makes this trade: `--bmax`/`--bmaxdivn`, and `--dcv`. By default, `centrifuge-build` will automatically search for the settings that yield the best running time without exhausting memory. This behavior can be disabled using the `-a`/`--noauto` option.

The indexer provides options pertaining to the "shape" of the index, e.g. `--offrate` governs the fraction of Burrows-Wheeler rows that are "marked" (i.e., the density of the suffix-array sample; see the original FM Index paper for details). All of these options are potentially profitable trade-offs depending on the application. They have been set to defaults that are reasonable for most cases according to our experiments. See Performance tuning for details.

The Centrifuge index is based on the FM Index of Ferragina and Manzini, which in turn is based on the Burrows-Wheeler transform. The algorithm used to build the index is based on the blockwise algorithm of Karkkainen.

## Command Line

Usage:

```
centrifuge-build [options]* --conversion-table <table_in> --taxonomy-tree <taxonomy_in> --name-table <table_in2> <reference_in> <cf_base>
```

### Main arguments

|  |  |
| --- | --- |
| ``` <reference_in> ``` | A comma-separated list of FASTA files containing the reference sequences to be aligned to, or, if `-c` is specified, the sequences themselves. E.g., `<reference_in>` might be `chr1.fa,chr2.fa,chrX.fa,chrY.fa`, or, if `-c` is specified, this might be `GGTCATCCT,ACGGGTCGT,CCGTTCTATGCGGCTTA`. |
| ``` <cf_base> ``` | The basename of the index files to write. By default, `centrifuge-build` writes files named `NAME.1.cf`, `NAME.2.cf`, and `NAME.3.cf`, where `NAME` is `<cf_base>`. |

### Options

|  |  |
| --- | --- |
| ``` -f ``` | The reference input files (specified as `<reference_in>`) are FASTA files (usually having extension `.fa`, `.mfa`, `.fna` or similar). |
| ``` -c ``` | The reference sequences are given on the command line. I.e. `<reference_in>` is a comma-separated list of sequences rather than a list of FASTA files. |
| ``` -a/--noauto ``` | Disable the default behavior whereby `centrifuge-build` automatically selects values for the `--bmax`, `--dcv` and [`--packed`] parameters according to available memory. Instead, user may specify values for those parameters. If memory is exhausted during indexing, an error message will be printed; it is up to the user to try new parameters. |
| ``` -p/--threads <int> ``` | Launch `NTHREADS` parallel search threads (default: 1). |
| ``` --conversion-table <file> ``` | List of UIDs (unique ID) and corresponding taxonomic IDs. |
| ``` --taxonomy-tree <file> ``` | Taxonomic tree (e.g. nodes.dmp). |
| ``` --name-table <file> ``` | Name table (e.g. names.dmp). |
| ``` --size-table <file> ``` | List of taxonomic IDs and lengths of the sequences belonging to the same taxonomic IDs. |
| ``` --bmax <int> ``` | The maximum number of suffixes allowed in a block. Allowing more suffixes per block makes indexing faster, but increases peak memory usage. Setting this option overrides any previous setting for `--bmax`, or `--bmaxdivn`. Default (in terms of the `--bmaxdivn` parameter) is `--bmaxdivn` 4. This is configured automatically by default; use `-a`/`--noauto` to configure manually. |
| ``` --bmaxdivn <int> ``` | The maximum number of suffixes allowed in a block, expressed as a fraction of the length of the reference. Setting this option overrides any previous setting for `--bmax`, or `--bmaxdivn`. Default: `--bmaxdivn` 4. This is configured automatically by default; use `-a`/`--noauto` to configure manually. |
| ``` --dcv <int> ``` | Use `<int>` as the period for the difference-cover sample. A larger period yields less memory overhead, but may make suffix sorting slower, especially if repeats are present. Must be a power of 2 no greater than 4096. Default: 1024. This is configured automatically by default; use `-a`/`--noauto` to configure manually. |
| ``` --nodc ``` | Disable use of the difference-cover sample. Suffix sorting becomes quadratic-time in the worst case (where the worst case is an extremely repetitive reference). Default: off. |
| ``` -o/--offrate <int> ``` | To map alignments back to positions on the reference sequences, it's necessary to annotate ("mark") some or all of the Burrows-Wheeler rows with their corresponding location on the genome. `-o`/`--offrate` governs how many rows get marked: the indexer will mark every 2^`<int>` rows. Marking more rows makes reference-position lookups faster, but requires more memory to hold the annotations at runtime. The default is 4 (every 16th row is marked; for human genome, annotations occupy about 680 megabytes). |
| ``` -t/--ftabchars <int> ``` | The ftab is the lookup table used to calculate an initial Burrows-Wheeler range with respect to the first `<int>` characters of the query. A larger `<int>` yields a larger lookup table but faster query times. The ftab has size 4^(`<int>`+1) bytes. The default setting is 10 (ftab is 4MB). |
| ``` --seed <int> ``` | Use `<int>` as the seed for pseudo-random number generator. |
| ``` --kmer-count <int> ``` | Use `<int>` as kmer-size for counting the distinct number of k-mers in the input sequences. |
| ``` -q/--quiet ``` | `centrifuge-build` is verbose by default. With this option `centrifuge-build` will print only error messages. |
| ``` -h/--help ``` | Print usage information and quit. |
| ``` --version ``` | Print version information and quit. |

# The `centrifuge-inspect` index inspector

`centrifuge-inspect` extracts information from a Centrifuge index about what kind of index it is and what reference sequences were used to build it. When run without any options, the tool will output a FASTA file containing the sequences of the original references (with all non-`A`/`C`/`G`/`T` characters converted to `N`s). It can also be used to extract just the reference sequence names using the `-n`/`--names` option or a more verbose summary using the `-s`/`--summary` option.

## Command Line

Usage:

```
centrifuge-inspect [options]* <cf_base>
```

### Main arguments

|  |  |
| --- | --- |
| ``` <cf_base> ``` | The basename of the index to be inspected. The basename is name of any of the index files but with the `.X.cf` suffix omitted. `centrifuge-inspect` first looks in the current directory for the index files, then in the directory specified in the `Centrifuge_INDEXES` environment variable. |

### Options

|  |  |
| --- | --- |
| ``` -a/--across <int> ``` | When printing FASTA output, output a newline character every `<int>` bases (default: 60). |
| ``` -n/--names ``` | Print reference sequence names, one per line, and quit. |
| ``` -s/--summary ``` | Print a summary that includes information about index settings, as well as the names and lengths of the input sequences. The summary has this format:   ``` Colorspace  <0 or 1> SA-Sample   1 in <sample> FTab-Chars  <chars> Sequence-1  <name>  <len> Sequence-2  <name>  <len> ... Sequence-N  <name>  <len> ```   Fields are separated by tabs. Colorspace is always set to 0 for Centrifuge. |
| ``` --conversion-table ``` | Print a list of UIDs (unique ID) and corresponding taxonomic IDs. |
| ``` --taxonomy-tree ``` | Print taxonomic tree. |
| ``` --name-table ``` | Print name table. |
| ``` --size-table ``` | Print a list of taxonomic IDs and lengths of the sequences belonging to the same taxonomic IDs. |
| ``` -v/--verbose ``` | Print verbose output (for debugging). |
| ``` --version ``` | Print version information and quit. |
| ``` -h/--help ``` | Print usage information and quit. |

# Getting started with Centrifuge

Centrifuge comes with some example files to get you started. The example files are not scientifically significant; these files will simply let you start running Centrifuge and downstream tools right away.

First follow the manual instructions to obtain Centrifuge. Set the `CENTRIFUGE_HOME` environment variable to point to the new Centrifuge directory containing the `centrifuge`, `centrifuge-build` and `centrifuge-inspect` binaries. This is important, as the `CENTRIFUGE_HOME` variable is used in the commands below to refer to that directory.

## Indexing a reference genome

To create an index for two small sequences included with Centrifuge, create a new temporary directory (it doesn't matter where), change into that directory, and run:

```
$CENTRIFUGE_HOME/centrifuge-build --conversion-table $CENTRIFUGE_HOME/example/reference/gi_to_tid.dmp --taxonomy-tree $CENTRIFUGE_HOME/example/reference/nodes.dmp --name-table $CENTRIFUGE_HOME/example/reference/names.dmp $CENTRIFUGE_HOME/example/reference/test.fa test
```

The command should print many lines of output then quit. When the command completes, the current directory will contain ten new files that all start with `test` and end with `.1.cf`, `.2.cf`, `.3.cf`. These files constitute the index - you're done!

You can use `centrifuge-build` to create an index for a set of FASTA files obtained from any source, including sites such as UCSC, NCBI, and Ensembl. When indexing multiple FASTA files, specify all the files using commas to separate file names. For more details on how to create an index with `centrifuge-build`, see the manual section on index building. You may also want to bypass this process by obtaining a pre-built index.

## Classifying example reads

Stay in the directory created in the previous step, which now contains the `test` index files. Next, run:

```
$CENTRIFUGE_HOME/centrifuge -f -x test $CENTRIFUGE_HOME/example/reads/input.fa
```

This runs the Centrifuge classifier, which classifies a set of unpaired reads to the the genomes using the index generated in the previous step. The classification results are reported to stdout, and a short classification summary is written to centrifuge-species\_report.tsv.

You will see something like this:

```
readID  seqID taxID     score   2ndBestScore    hitLength   numMatches
C_1 gi|7     9913      4225 4225        80      2
C_1 gi|4     9646      4225 4225        80      2
C_2 gi|4     9646      4225 4225        80      2
C_2 gi|7     9913      4225 4225        80      2
C_3 gi|7     9913      4225 4225        80      2
C_3 gi|4     9646      4225 4225        80      2
C_4 gi|4     9646      4225 4225        80      2
C_4 gi|7     9913      4225 4225        80      2
1_1 gi|4     9646      4225 0       80      1
1_2 gi|4     9646      4225 0       80      1
2_1 gi|7     9913      4225 0       80      1
2_2 gi|7     9913      4225 0       80      1
2_3 gi|7     9913      4225 0       80      1
2_4 gi|7     9913      4225 0       80      1
2_5 gi|7     9913      4225 0       80      1
2_6 gi|7     9913      4225 0       80      1
```
