# Supplementary material for: Centrifuge: rapid and sensitive classification of metagenomic sequences
Source: Genome Res. 2016 Dec;26(12):1721–9. doi: 10.1101/gr.210641.116 (PMC5131823; doi:10.1101/gr.210641.116)
Supplement: Supplemental Material [file supp_gr.210641.116_Supplemental_Figure_S1.docx]

Supplemental Figure S1. Comparison of log-scaled true abundance and Centrifuge abundance estimates of genomes based on 10 million simulated reads (Pearson’s correlation coefficients: 0.919 at the species level, 0.986 at the genus level, 0.993 at the family level, 0.993 at the order level, 0.996 at the class level, and 0.999 at the phylum level).
