# Supplementary material for: Centrifuge: rapid and sensitive classification of metagenomic sequences
Source: Genome Res. 2016 Dec;26(12):1721–9. doi: 10.1101/gr.210641.116 (PMC5131823; doi:10.1101/gr.210641.116)
Supplement: Supplemental Material [file supp_gr.210641.116_Supplemental_Figure_S2.docx]

Supplemental Figure S2. Sensitivity and precision comparison of Centrifuge and Kraken for 530 real sequencing data sets at the genus level. The left plot shows the sensitivity and precision comparison from 0 to 100%. The right plot magnifies the sensitivity and the precision comparison between 92.5 to 100% and between 95 to 100%, respectively, where ~50% of the datasets are located.
