# Supplementary material for: Centrifuge: rapid and sensitive classification of metagenomic sequences
Source: Genome Res. 2016 Dec;26(12):1721–9. doi: 10.1101/gr.210641.116 (PMC5131823; doi:10.1101/gr.210641.116)
Supplement: Supplemental Material [file supp_gr.210641.116_Supplemental_Methods.docx]

**Centrifuge: rapid and sensitive classification of metagenomic sequences**

**Daehwan Kim, Li Song, Florian P. Breitwieser, and Steven L. Salzberg**

*Supplemental Methods*

*Optimization of FM-index to address the classification problem*

In contrast to alignment where mapped locations are reported (e.g. the 26,987,299^th^ base on chromosome 14), classifiers need only to label a read with a name at the genome level, species level, or higher taxonomic rank. Instead of using 4 or 8 bytes to store an exact location, we just use two bytes to store the genome or species ID. This decreases the index size by >20%. We also developed a parallelized version of FM index building, which allowed us to build our Centrifuge index for the NCBI *nt* database in only 29 hours using 16 CPU cores and 280 GB of RAM, a process that previously required over two weeks.

*Evaluation of Centrifuge using two alternative settings*

Based on the same Mason simulated reads used in the main text, we also ran Centrifuge to perform additional evaluations by changing the settings, switching from compressed to uncompressed bacterial sequences, and also changing the FM index parameters to use a larger internal table as well as storing one genome ID per four bases (instead of per 16 bases), which together enables fast access in the FM index and reduces the number of operations in the FM index. In the first case, sensitivity and precision using the uncompressed sequences remained nearly the same compared to compressed sequences; i.e., compression did not affect sensitivity. In the second case, with an FM index 2.3 times larger than the default mode, processing speed increased by 67%, which is nearly as fast as Kraken.

*Performance of Centrifuge in classifying divergent genomes*

To estimate how Centrifuge performs in classifying reads from genomes divergent from those in the database, we randomly chose 10% of the genomes of each genus (with at least two genomes) to form a narrower database. We then used Centrifuge to classify simulated reads (referred to in Table 2 of the main text) from genomes not included in the smaller database. At the genus level, while Centrifuge’s sensitivity is low, 56.3%, precision is high, 95.5%.

*Building the Centrifuge index*

As part of the Centrifuge package available at the Centrifuge website (http://ccb.jhu.edu/software/centrifuge), we provide scripts for downloading the microbial reference genomes, contaminants and the human genome, and for building the database. The microbial genome sequences are post-processed with NCBI’s dustmasker to mask low-complexity regions [[1](#_ENREF_1)]. Low-complexity regions otherwise can lead to spurious matches. Furthermore the database includes the latest release of the UniVec database (<ftp://ftp.ncbi.nlm.nih.gov/pub/UniVec>), the EmVec database (<ftp://ftp.ebi.ac.uk/pub/databases/emvec>), and other artificial sequences.

*Simulating reads using Mason (version: 0.1)*

The 4,078 bacterial, 200 archaeal, human, and viral genomes are available for download at <ftp://ftp.ccb.jhu.edu/pub/infphilo/centrifuge/downloads/centrifuge-suppl/data.tar.gz>.

The compressed file, data.tar.gz, includes the following files:

seqid+gid_to_taxid.map: the map of the sequence id and gid to taxonomy id

and the following folders:

taxonomy: taxonomy information such as taxonomy tree and genome names

bacteria+archaea: the sequence we used to build the index for Centrifuge and other tools

bacteria_sim10K: the simulated data set and its truth assignment. Though the name is simplified for brevity, the bacteria_sim10K.fa also contains reads from both bacteria and archaea.

1. Build the reference genome sim_ref.fa from which we simulate reads by concatenating the *.fna files in the folder bacteria+archaea/bacteria and bacteria+archaea/archaea.

2. Run Mason to simulate 20 million 100-bp reads with a per-base error rate of 3%:

/path/to/mason illumina -i -n 100 -N 20000000 -pi 0.005 -pd 0.005 -pmm 0.02 -pmmb 0.01 -pmme 0.06 -o bacteria_sim20M.fa sim_ref.fa

3. Remove the reads from the genomes whose taxonomic id has no corresponding species or genus unit, which results in 19,851,369 reads left.

4. Randomly sample 10 million reads without replacement from the reads retained in the above step. This resulting file, bacteria_sim10M.fa, is used for the speed tests of Centrifuge and Kraken.

5. Randomly sample 10 thousand reads without replacement from bacteria_sim10M.fa to bacteria_sim10K.fa. This file is used for the accuracy tests of Centrifuge, Kraken, and MegaBLAST.

*Running Centrifuge, Kraken, and MegaBLAST*

Centrifuge (version: 1.0.1-beta):

perl /path/to/centrifuge-compress.pl B_only taxonomy/ -map seqid_to_taxid.map –o centrifuge_db

/path/to/centrifuge-build –p 4 –conversion-table centrifuge_db.map –taxonomy-tree taxonomy/nodes.dmp –name-table taxonomy/names.dmp centrifuge_db.fa centrifuge_db

/path/to/centrifuge --no-abundance -f -x centrifuge_db –U bacteria_sim10K.fa

Kraken (version: 0.10.5-beta):

/path/to/kraken-build --build --db=kraken_db

/path/to/kraken --db kraken_db/ --fasta-input bacteria_10K.fa

MegaBLAST (version: 2.2.30+):

We concatenate all the *.fa and *.fna files in the bacteria+archaea folder and store the sequences to bacteria+archaea_dustmask.fa after running dustmask.

/path/to/makeblastdb –in bacteria+archaea_dustmask.fa –dbtype nucl –out megablast.db

/path/to/makembindex –input bacteria+archaea_dustmask.fa –output megablast.db

/path/to/blastn -query bacteria_sim10K.fa –db megblast.db -index_name megablast.db -out megablast.out

*Downloading whole-genome sequencing data sets of 530 isolated bacterial genomes in the Short Read Archive*

On the NCBI website at <http://www.ncbi.nlm.nih.gov>,

We used the following query (shown in blue) in the search field, and clicked “Search” button:

("2015/1/1"[PDAT] : "2015/9/23"[PDAT]) AND ("Bacteria"[Organism] OR "Bacteria"[Organism] OR "bacteria"[All Fields]) AND ("biomol dna"[Properties] AND "strategy wgs"[Properties])

On the result webpage, we followed the “SRA” link under “Genomes”.

In the list of search results, we clicked “Send to:”, chose “File” and format “Runinfo” to create and download a file providing a summary of the experiments.

Then we selected the experiments using HiSeq and MiSeq technology with spots (reads) between 200,000 and 2,000,000.

Centrifuge takes 8 hours 34 minutes, Kraken takes 7 hours 12 minutes, and MegaBLAST takes 2 hours 36 minutes for building their respective indexes of ~4,300 prokaryotes.  Centrifuge and Kraken allow for faster index building when using multiple CPU cores.

## References

1. Morgulis A, Gertz EM, Schaffer AA, Agarwala R: **A fast and symmetric DUST implementation to mask low-complexity DNA sequences.** *J Comput Biol* 2006, **13:**1028-1040.
