# Supplementary material for: Centrifuge: rapid and sensitive classification of metagenomic sequences
Source: Genome Res. 2016 Dec;26(12):1721–9. doi: 10.1101/gr.210641.116 (PMC5131823; doi:10.1101/gr.210641.116)
Supplement: Supplemental Material [file supp_gr.210641.116_Supplemental_Table_S1.docx]

Supplemental Table S1. Centrifuge alternative setting comparisons

| Settings | Genus sensitivity | Genus  precision | Speed (reads/min) | Memory usage (GB of RAM) |
| --- | --- | --- | --- | --- |
| Default | 93.1 | 99.6 | 563,380 | 4.2 |
| Index based on uncompressed sequences | 93.7 | 99.6 | 369,231 | 6.9 |
| FM index (with higher memory requirements) | 93.1 | 99.6 | 943,396 | 9.6 |
